# Electrolyte‐Assisted Structure Reconstruction Optimization of Sn‐Zn Hybrid Oxide Boosts the Electrochemical CO_2_‐to‐HCOO^−^ Conversion

**DOI:** 10.1002/advs.202407019

**Published:** 2024-08-19

**Authors:** Jinxian Feng, Chunfa Liu, Lulu Qiao, Keyu An, Sen Lin, Weng Fai Ip, Hui Pan

**Affiliations:** ^1^ Institute of Applied Physics and Materials Engineering University of Macau Macao SAR 999078 China; ^2^ State Key Laboratory of Photocatalysis on Energy and Environment College of Chemistry Fuzhou University Fuzhou 350108 China; ^3^ Department of Physics and Chemistry Faculty of Science and Technology University of Macau Macao SAR 999078 China

**Keywords:** CO_2_ reduction, electrolyte, formate, Sn‐Zn oxide, surface reconstruction

## Abstract

Electrolyte plays crucial roles in electrochemical CO_2_ reduction reaction (e‐CO_2_RR), yet how it affects the e‐CO_2_RR performance still being unclarified. In this work, it is reported that Sn‐Zn hybrid oxide enables excellent CO_2_‐to‐HCOO^−^ conversion in KHCO_3_ with a HCOO^−^ Faraday efficiency ≈89%, a yield rate ≈0.58 mmol cm^−2^ h^−1^ and a stability up to ≈60 h at −0.93 V, which are higher than those in NaHCO_3_ and K_2_SO_4_. Systematical characterizations unveil that the surface reconstruction on Sn‐Zn greatly depends on the electrolyte using: the Sn‐SnO_2_/ZnO, the ZnO encapsulated Sn‐SnO_2_/ZnO and the Sn‐SnO_2_/Zn‐ZnO are reconstructed on the surface by KHCO_3_, NaHCO_3_ and K_2_SO_4_, respectively. The improved CO_2_‐to‐HCOO^−^ performance in KHCO_3_ is highly attributed to the reconstructed Sn‐SnO_2_/ZnO, which can enhance the charge transportation, promote the CO_2_ adsorption and optimize the adsorption configuration, accumulate the protons by enhancing water adsorption/cleavage and limit the hydrogen evolution. The findings may provide insightful understanding on the relationship between electrolyte and surface reconstruction in e‐CO_2_RR and guide the design of novel electrocatalyst for effective CO_2_ reduction.

## Graphical abstract

Electrolyte plays an essential role in electrocatalysis. The Cu‐incorporated SnO_2_/ZnO reconstructs to Sn‐SnO_2_/ZnO in KHCO_3_, enabling better CO_2_‐to‐HCOO^−^ conversion performance than those in NaHCO_3_ and K_2_SO_4_. The reconstructed Sn‐SnO_2_/ZnO has positively charged surface that enhances the electron/charge transportation, promotes the CO_2_ adsorption, accumulates the protons by enhancing water adsorption/cleavage and alleviates the hydrogen evolution, so benefit for HCOO^–^ generation.

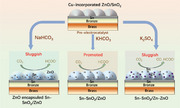

## 1 Introduction

The electrochemical CO_2_ reduction reaction (e‐CO_2_RR) driven by electricity from sustainable power sources is one of the efficient pathways toward carbon neutrality,^[^ ^1^ ^]^ and store the excess electricity in stable chemical forms.^[^ ^2^ ^]^ Among all the possible productions of e‐CO_2_RR, formic acid or formate (HCOO^−^) can be directly used in fuel cells or as hydrogen carriers.^[^ ^3^ ^]^ Besides, HCOO^−^ is also one of the most important feedstocks for pharmaceutical, fine chemical industry and food additives.^[^ ^4^ ^]^ Therefore, it is a “killing three birds by one stone” strategy for producing HCOO^−^ by e‐CO_2_RR. Up to now, the cost‐effective and ecofriendly elements, like Sn, Bi, Zn, and Cu, as well as their compounds, are considered as compositions of high‐efficient CO_2_‐to‐HCOO^−^ electrocatalysts.^[^ ^3^, ^5^ ^]^ Nevertheless, despite of the rapid development in the past decades, there are still a lot of challenges for the HCOO^−^ generation by e‐CO_2_RR to be practically applied, such as low selectivity and poor stability.^[^ ^6^ ^]^

Recently, it has been reported that the electrolytes are critical to the activity, selectivity and stability of e‐CO_2_RR electrocatalysts.^[^ ^7^ ^]^ For example, Wu et al. found that different cations and anions affected the structure of Helmholtz layer on the electrocatalyst, leading to varieties activity and selectivity of CO_2_‐to‐HCOO^−^ conversion.^[^ ^8^ ^]^ Monterio et. al. reported that the Cs^+^ ion could change the micro environment close to the surface and therefore tune the e‐CO_2_RR efficiency.^[^ ^9^ ^]^ As we know, the as‐prepared electrocatalyst (pre‐electrocatalyst) reconstructed in the electrocatalytic process more or less. The interactions between electrolyte and pre‐electrocatalyst may trigger different reconstructed structure, thus affecting the electrocatalytic performance.^[^ ^10^ ^]^ Unfortunately, the mechanisms on how the electrolyte affects the surface reconstruction of electrocatalyst and improves the CO_2_‐to‐HCOO^−^ conversion are still unclarified.

In this work, we fabricate Cu‐incorporated SnO_2_/ZnO (B‐Sn‐A) as pre‐electrocatalyst based on low‐cost commercial brass H62 (B) through combined electrodeposition and heat annealing. We find that reconstructed Sn‐SnO_2_/ZnO, ZnO encapsulated Sn‐SnO_2_/ZnO and Sn‐SnO_2_/Zn‐ZnO are introduced by using KHCO_3_, NaHCO_3_, and K_2_SO_4_ as electrolyte, respectively. Typically, the reconstructed Sn‐SnO_2_/ZnO on B‐Sn‐A in KHCO_3_ achieves the highest catalytic performance for the CO_2_‐to‐HCOO^−^ conversion, including a HCOO^‐^ Faraday efficiency (FE) of ≈89%, a yield rate of ≈0.58 mmol cm^−2^ h^−1^ and a partial current density (j_HCOO‐_) of 31.14 mA cm^−2^ at −0.93 V versus RHE (reversible hydrogen electrode), which are much higher than those of most reported electrocatalysts. Systematically in situ and ex situ experiments show that the Sn and Zn sites of the reconstructed Sn‐SnO_2_/ZnO in KHCO_3_ are positively charged, that favorable for CO_2_‐to‐HCOO^−^ conversion kinetics due to these following issues: i) the charge/electron transportation is accelerated; ii) the CO_2_ adsorption is improved and the CO_2_ adsorption configuration is optimized; iii) the proton concentration is promoted through the water adsorption/cleavage enhancement and the HER prohibition.

## 2 Results and Discussion

### 2.1 Structural Characterizations of Pre‐Electrocatalyst

We performed systematically electronic microscopy and spectroscopy methods to study the structure and composition of the B‐Sn‐A pre‐electrocatalyst. The SEM images show that B‐Sn‐A is consisted by various nanoparticles with ≈100 nm in sizes (Figure S1a, Supporting Information), and the EDS shows the Cu, Sn, Zn, and O signals co‐exist (Figure S1b, Supporting Information). The XRD pattern shows the characteristic peaks of brass (JCPDS 50–1333), bronze (JCPDS 45–1488), ZnO (JCPDS 36–1451), SnO_2_ (JCPDS 41–1445) and Sn (JCPDS 04–0672) (Figure S2, Supporting Information). The Raman spectroscopy shows the characteristic peaks of Zn‐O, Cu‐O and Sn‐O clearly (Figure S3, Supporting Information),^[^ ^11^ ^]^ while the XPS O 1s spectra show the metal‐O and metal‐OH signals, indicating the metal oxide exist (Figure S4a, Supporting Information).^[^ ^12^ ^]^ The XPS Zn 2p, Cu 2p, and Sn 3d_3/2_ spectra show the characteristic binding energy signals that can be assigned to Zn^2+^ of Zn oxide,^[^ ^13^ ^]^ Cu^2+^ of Cu oxide,^[^ ^14^ ^]^ and Sn^4+^ of Sn oxide,^[^ ^15^ ^]^ respectively (Figure S4b–d, Supporting Information). Therefore, we infer that B‐Sn‐A is composed of Cu‐incorporated ZnO/SnO_2_ hybrid oxide.

### 2.2 e‐CO_2_RR Performances

**Figure 1:**
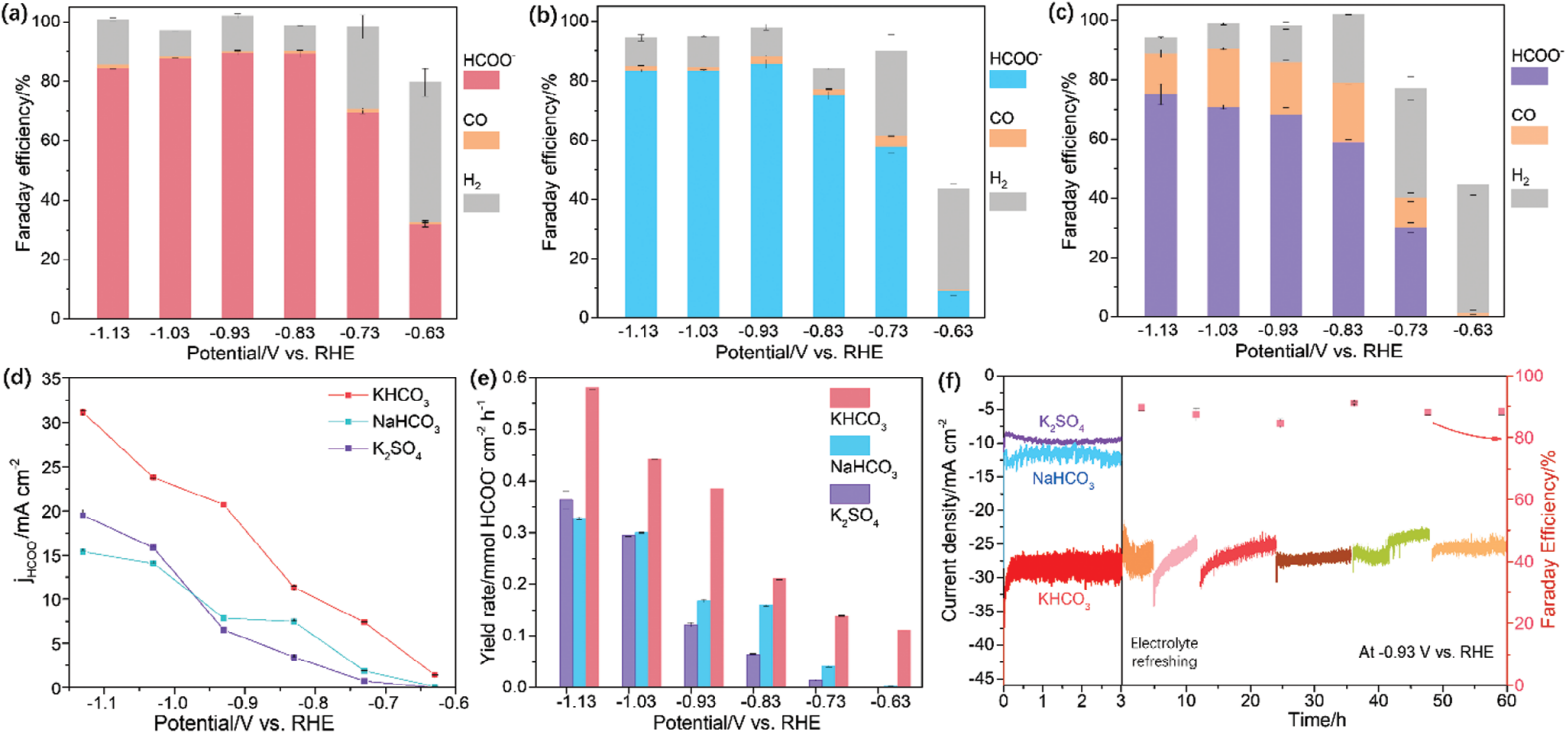
Product Distributions of B‐Sn‐A in: a) 0.5 m KHCO_3_, b) 0.5 m NaHCO_3_, and c) 0.25 m K_2_SO_4_. d) j_HCOO‐_ and e) HCOO^−^ yield rates of B‐Sn‐A at different potentials using KHCO_3_, NaHCO_3_ and K_2_SO_4_ as electrolytes. f) 3‐h e‐CO_2_RR of B‐Sn‐A in different electrolytes and 60 h ADT measurement with six on‐and‐off galvanostatic tests at −0.93 V in KHCO_3_.

**Figure 2:**
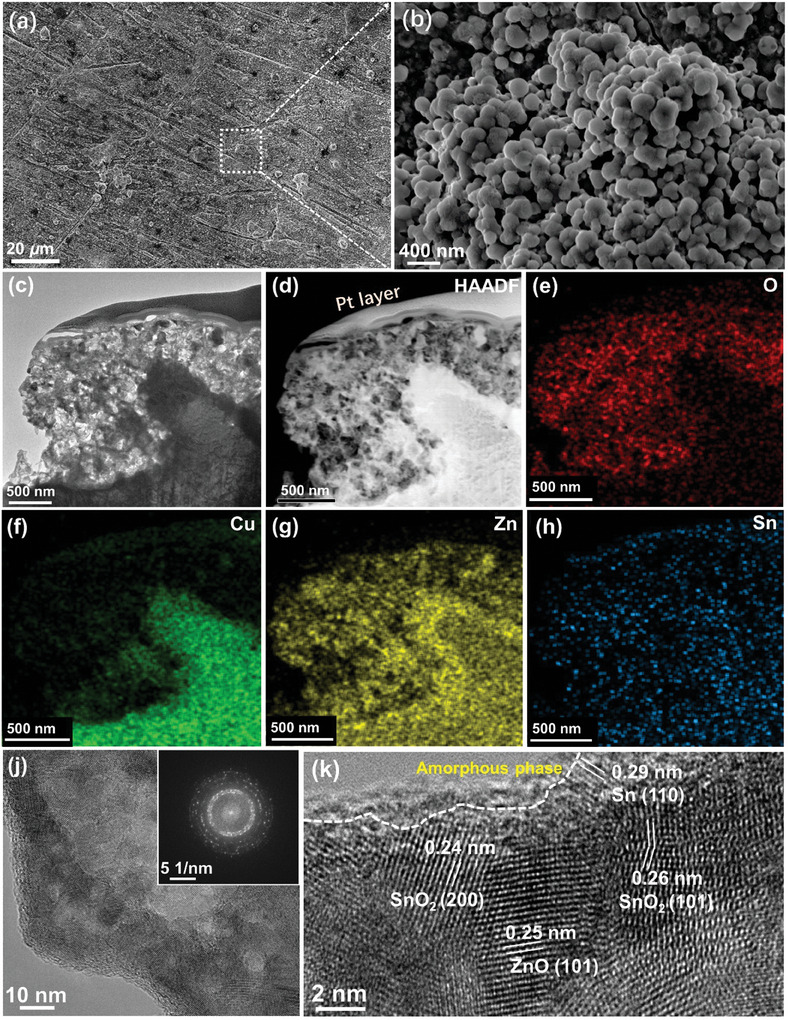
B‐Sn‐A after the e‐CO_2_RR test in KHCO_3_: a,b) SEM images. c) Cross‐section TEM image. d) HAADF image and e–h) EDS mappings. j) TEM image (Inset: FFT image). k) HRTEM image.

The e‐CO_2_RR performance was measured in CO_2_‐saturated 0.5 m KHCO_3_, 0.5 m NaHCO_3_, and 0.25 m K_2_SO_4_ solutions, respectively. The liquid and gaseous products were examined by HPLC and on‐line GC, respectively. Only CO and H_2_ can be detected in the gas phase (Figures S5–S7, Supporting Information). The H_2_ FEs are suppressed below 20% when the potential is negative than −0.83 V (Figure 2a–c). The CO FEs in KHCO_3_ and NaHCO_3_ are <5% when the potential is negative than −0.83 V, while that in K_2_SO_4_ is >15% (Figure 2a–c). The UV–vis spectra show HCOO^−^ generation in e‐CO_2_RR (Figure S5, Supporting Information), and the HPLC chromatograms show that only HCOO^−^ can be detected (Figure S6, Supporting Information). The B‐Sn‐A in KHCO_3_ and NaHCO_3_ shows a HCOO^−^ FE of >80% at a volage range of −0.83–1.13 V, where the highest HCOO^−^ FE are 89% and 87.5% at −0.93 V, respectively (**Figure** **1**a,b). In contrast, the HCOO^−^ FE for B‐Sn‐A in K_2_SO_4_ only achieves 68% at −0.93 V (Figure 1c). These results underscore significant influence of electrolyte on FEs of products: the carbonate electrolytes prefer for HCOO^−^ generation, while K_2_SO_4_ is more prone to the CO_2_‐to‐CO conversion. Meanwhile, B‐Sn‐A in KHCO_3_ displays larger j_HCOO‐_ and HCOO^−^ yield rate than those in NaHCO_3_ and K_2_SO_4_ (Figure 1d,e; Figures S7 and S8, Supporting Information). Additionally, the HCOO^−^ FE, yield rates and j_HCOO‐_ of B‐Sn‐A are higher than those of B, B‐A, and B‐Sn in KHCO_3_ (Figure S10, Supporting Information). To evaluate the long‐term stability, accelerate degradation test (ADT) was conducted. B‐Sn‐A shows higher current density in KHCO_3_ than that in NaHCO_3_ and K_2_SO_4_ in 3‐h chronoamperometry test at −0.93 V (Figure 1f). Moreover, B‐Sn‐A shows excellent stability up to ≈60 h ADT test at −0.93 V with six on and off cycles in KHCO_3_, as demonstrated by the negligible reduction in the current density and FE value after electrolyte refreshing followingly (Figure 1f).

To exemplify the electrochemical performance under simulated industrial condition, we measured the e‐CO_2_RR performance in a homemade two‐electrode flow cell. We can see that the B‐Sn‐A attains remarkable FE HCOO^−^ of 69.3%, 82.0%, 84.2%, and 75.4% at 25, 50, 100, and 150 mA cm^−2^, respectively. Impressively, the FE HCOO^−^ are not <≈80% with five on‐and‐off ADT cycle tests at 50 and 100 mA cm^−2^, indicating the superior stability in the industrial condition (Figure S11, Supporting Information). Most importantly, the B‐Sn‐A outperforms the majority of state‐of‐the‐art CO_2_‐to‐HCOO^−^ conversion electrocatalysts reported to date in KHCO_3_ (Table S1, Supporting Information).

### 2.3 Structural Characterizations After a 3‐h Test

To reveal the origin for the high performance, the systematical structure characterizations were carried out. The SEM images show that the surface of B‐Sn‐A after a 3‐h test in KHCO_3_ is composed of nanoparticles with a size of ≈50 nm (**Figure** **2**a), while the surface of the sample tested in NaHCO_3_ appears as hierartical nanoparticles (≈2 µm) aggregated by particles with various diameters (≈100 nm) (Figure S13a,b, Supporting Information). Differently, the B‐Sn‐A after the 3‐h test in K_2_SO_4_ shows randomly oriented nanosheets (≈200 nm) on surface (Figure S14a,b, Supporting Information). The EDS mappings show the existence of Cu, Sn, Zn and O signals that may come from substrate or oxide layer (Figures S12 and S14, Supporting Information). The TEM, the high‐angle annular dark‐field scanning transmission electron microscopy images (HAADF‐STEM) and the corresponding EDS mappings on the cross section of B‐Sn‐A after the 3‐h test in KHCO_3_ clearly show the layered distribution of Cu, Sn, Zn and O, where the Pt is the protective layer used in the FIB process (Figure 2c,d). The O distribution indicates that the surface oxide layer is about 500–1000 nm in thickness (Figure 2d). Notably, different from Zn and Sn, the Cu signal becomes weaker gradually from bulk to surface, suggesting a Cu‐poor phase on surface (Figure 2e–h). The HAADF‐STEM EDS mappings of B‐Sn‐A after the tests in NaHCO_3_ and K_2_SO_4_ suggest the co‐existence of Zn, Sn, and O as well (Figures S13d,d1–d3 and S14d,d1–d3, Supporting Information). The fast Fourier transition (FFT) patterns confirm the polycrystal structure of all the samples (Inset in Figure 2j; Figures S13e and S15e, Supporting Information). The high‐resolution transmission electronic microscopy (HRTEM) images of all the samples exhibit the lattice planes of Sn, ZnO, and SnO_2_, suggesting the Sn‐ZnO‐SnO_2_ hybridization of B‐Sn‐A after test in KHCO_3_, NaHCO_3_, and K_2_SO_4_ (Figure 2k; Figures S13f1–f3 and S15f1–f3, Supporting Information), matching the EDS mapping well. Notably, a 2 nm‐thick amorphous layer on the surface of B‐Sn‐A tested in KHCO_3_ can be observed, which should play crucial role in e‐CO_2_RR electrocatalysis (Figure 2k).

**Figure 3:**
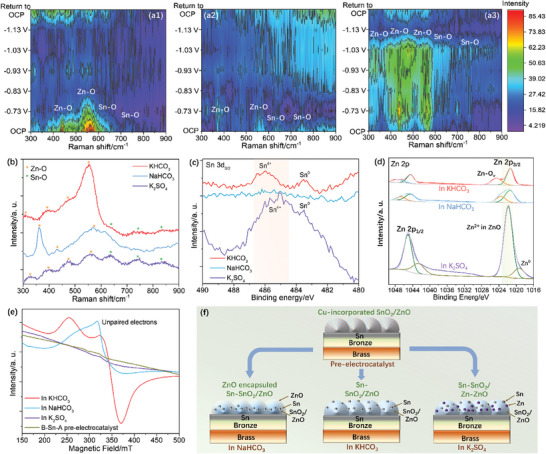
In situ Raman spectra of B‐Sn‐A measured at different potentials in: a1) KHCO_3_, a2) NaHCO_3_, and a3) K_2_SO_4_. b) Raman spectra. c) Sn 3d_5/2_ and d) Zn 2p XPS spectra of B‐Sn‐A surface. e) Quasi in situ ESR spectra of B‐Sn‐A after the tests in different electrolytes. f) Scheme of surface reconstructions.

The time‐dependent Zn, Cu and Sn concentrations in different electrolytes were measured to elucidate the dynamic change of elemental composition. For all the samples, Zn, Sn, and Cu concentrations increase at the time up to 10 min, then drop sharply and retain stable after 30 min (Figure S16, Supporting Information), indicating the elements leaching takes the main place at the first 10 min, then the redeposition becomes the dominant gradually after 10 min. Notably, the element concentrations in electrolytes after 20 min follow: Zn >> Sn > Cu, suggesting Zn is more prone to dissolve and redeposit. Interestingly, in different electrolyte, the Zn concentration follows: NaHCO_3_ > K_2_SO_4_ ≥ KHCO_3_, suggesting the Na^+^ and HCO_3_^−^ are more prefer for Zn dissolution and redeposition. Moreover, the cyclic voltammetry (CV) curves show that the intensities of Sn oxidation peak after 3‐h test at different potentials in KHCO_3_ are higher than those of NaHCO_3_ and K_2_SO_4_, underscoring Sn redeposition shall be easily (Figure S17, Supporting Information). The in situ Raman spectroscopy was utilized to study the surface state in e‐CO_2_RR further. We can see that the characteristic peaks of metal oxides are diminished and vanished eventually in the electrolysis at potential interval −0.73–1.13 V in KHCO_3_ and NaHCO_3_, suggesting the metal oxide convert to metal (**Figure** **3**a1,a2).^[^ ^16^ ^]^ However, there are still some Zn‐O and Sn‐O characteristic peaks remained though their characteristic peak diminished at a voltage range of −0.73–1.13 V in K_2_SO_4_, indicating that metal oxides keep partially (Figure 3a3). Therefore, the e‐CO_2_RR active phase shall be the zero‐valence metal in KHCO_3_ and NaHCO_3_, while the hybrid metal/metal oxide shall be the e‐CO_2_RR active phase in K_2_SO_4_.

The reconstructed structures are studied by XRD, Raman, XPS and ESR further. XRD patterns reveal that B‐Sn‐A after the 3‐h tests in KHCO_3_, NaHCO_3_ and K_2_SO_4_ are composed of brass (JCPDS 50–1333), bronze (JCPDS 45–1488), ZnO (JCPDS 36–1451), and SnO_2_ (JCPDS 41–1445) as well as Sn (JCPDS 04–0673) (Figure S18, Supporting Information). The Raman spectra show the characteristic peaks of Zn‐O and Sn‐O after the tests in KHCO_3_ and K_2_SO_4_ (Figure 3b).^[^ ^17^ ^]^ In contrast, only the Zn‐O characteristic peaks can be observed after the test in NaHCO_3_ (Figure 3b).^[^ ^6^, ^17^, ^18^ ^]^ Those results confirm the Cu seldomly appear on the surface on all the sample, while only the Zn oxide presence after test in NaHCO_3_. We can see that the Zn‐O characteristic peaks of the sample after tests in different electrolytes exhibit shape changing and location shifts, suggesting the crystallinities and the bonding states of the reconstructed structures are differen.^[^ ^19^, ^20^, ^21^, ^22^ ^]^ The XPS was utilized to study the valence and surface electronic states. We can see that the XPS O 1s spectra show the peaks at 530, 531, 532, and 533 eV, which should be ascribed to O in metal−O, metal−OH, physical and chemical adsorption water molecules (Figures S19a, S20a, and S21a, Supporting Information).^[^ ^15^, ^23^ ^]^ The XPS Sn 3d_3/2_ spectra show that the Sn exhibits Sn^4+^ and Sn^0^ of B‐Sn‐A in KHCO_3_ and K_2_SO_4_ (Figure 3c),^[^ ^19^ ^]^ but negligible Sn signal can be observed on the surface of the B‐Sn‐A after test in NaHCO_3_ (Figure 3c), coincident well with the Raman spectra (Figure 3b). The binding energy of Sn^4+^ of B‐Sn‐A in KHCO_3_ is positively shifted compared with that in K_2_SO_4_ (Figure 3c), indicating decreased electron density on the Sn sites.^[^ ^19^, ^20^ ^]^ The Zn 2p fine spectra show the existence of Zn^2+^ of ZnO in all the samples.^[^ ^23^ ^]^ Notably, for the B‐Sn‐A after tests in KHCO_3_ and NaHCO_3_, the Zn^2+^ in vicinity of O vacancy (O_v_) (Zn‐O_v_) is observed,^[^ ^21^ ^]^ while the Zn^0^ presents after the test in K_2_SO_4_ (Figure 3d).^[^ ^14^, ^22^ ^]^ The binding energy of Zn^2+^ in ZnO follow the trend: KHCO_3_ < NaHCO_3_ < K_2_SO_4_, and the binding energy of Zn‐O_v_ of B‐Sn‐A in KHCO_3_ is higher than that in NaHCO_3_ (Figure 3d), underscoring the increased positive charge on the Zn sites and the O_v_ of the reconstructed B‐Sn‐A after 3‐h test in KHCO_3_. Additionally, the XPS Cu 2p spectra of all the samples show negligible Cu on the surfaces (Figures S19d, S20d, and S21d, Supporting Information), suggesting the Cu plays negligible role in e‐CO_2_RR, aligned well with the XRD, Raman and EDS results.

The quasi in situ ESR was utilized to study the unpaired electrons (Figure 3e). We can see that the B‐Sn‐A after 3‐h tests in KHCO_3_ and NaHCO_3_ show stronger ESR peaks than pre‐electrocatalyst, suggesting the increased unpaired electrons.^[^ ^10^ ^]^ For the B‐Sn‐A tests in KHCO_3_ and NaHCO_3_, the strong ESR peaks at B = 330 mT are originated from the unpaired electrons of O_v_.^[^ ^24^ ^]^ Typically, for the B‐Sn‐A after the test in KHCO_3_, a peak of lattice electron trapping sites in ZnO or SnO_2_ (like Zn^+^, Sn^+^) present at B = 250 mT can also be observed (Figure 3e),^[^ ^24b^ ^]^ higher than that after test in NaHCO_3_, indicating the reconstructed B‐Sn‐A in KHCO_3_ is unpaired electron enriched. On the contrary, the B‐Sn‐A after 3‐h test in K_2_SO_4_ shows negligible ESR signal, indicating few unpaired electrons.

Combining the XRD, Raman scatting, XPS and ESR results, we can conclude that the Sn‐SnO_2_/ZnO, the ZnO encapsulated Sn‐SnO_2_/ZnO, and the Sn‐SnO_2_/Zn‐ZnO are reconstructed on B‐Sn‐A in KHCO_3_, NaHCO_3_ and K_2_SO_4_, respectively, through element dissolution‐redeposition (Figure 3f). Also, the KHCO_3_ and NaHCO_3_ introduce the Zn‐O_v_, and the K_2_SO_4_ triggers Zn° formation in the reconstructed structures. It is also evidenced that the KHCO_3_ makes the increased positive charge on the Zn and Sn sites of the reconstructed B‐Sn‐A. Meanwhile, we also find that the KHCO_3_ introduces more unpaired electrons and lattice electron trapping sites in the reconstructed structures.^[^ ^25^ ^]^ All these results elucidate that the unique electronic state of the reconstructed structure triggered by KHCO_3_ shall hold the key to the e‐CO_2_RR improvement.

### 2.4 Mechanism

**Figure 4:**
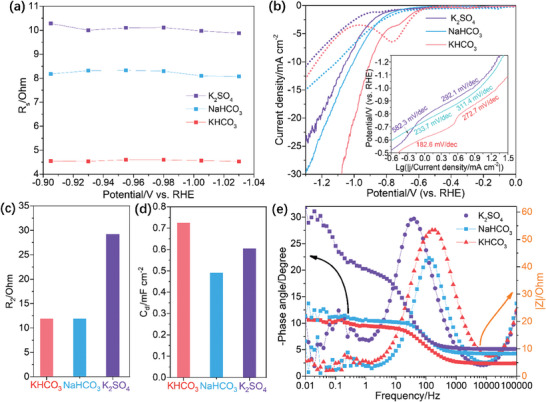
a) Plots of potential‐dependent R_s_. b) LSV curves and Tafel slopes (Insert). Column diagram of c) R_2_ and d) C_dl_ of B‐Sn‐A. e) Bode phase angle‐frequency and |Z|‐frequency plots measured at −0.93 V of B‐Sn‐A in CO_2_‐saturated KHCO_3_, NaHCO_3_ and K_2_SO_4_.

The LSV, Tafel analysis and operando EIS were conducted to elucidate the charge and mass transfer kinetics in e‐CO_2_RR, and proving that the B‐Sn‐A in KHCO_3_ can reduce the charge and mass transportation, some evidences are listed: i) The solution resistances (R_s_) of B‐Sn‐A in different electrolytes follow the trend: KHCO_3_ < NaHCO_3_ < K_2_SO_4_, indicating the KHCO_3_ can promote the mass and electron transportation at the electrolyte/electrocatalyst interface (**Figure** **4**a; Figure S23, Supporting Information).^[^ ^26^ ^]^ ii) The LSV curves of the B‐Sn‐A measured in CO_2_‐saturated KHCO_3_ show much higher current densities than those in NaHCO_3_ and K_2_SO_4_ consistently (Figure 4b), and the B‐Sn‐A in CO_2_‐saturated KHCO_3_ shows lower Tafel slopes than that in NaHCO_3_ and K_2_SO_4_, suggesting the promoted electron transfer of e‐CO_2_RR (insert in Figure 4b).^[^ ^27^ ^]^ iii) The KHCO_3_ and NaHCO_3_ show smaller surface ion transfer resistance (R_2_) than K_2_SO_4_, underscoring more robust ion transfer in bicarbonate electrolytes (Figure 4c). iv) The C_dl_ of the B‐Sn‐A in KHCO_3_ is larger, indicating the reconstructed structure of B‐Sn‐A in KHCO_3_ possessed enriched active sites for CO_2_ adsorption and activation (Figure 4d).^[^ ^28^ ^]^ v) The peak at 10–1000 Hz of the Bode ‐phase angle‐frequency plots measured at −0.93 V in different electrolytes follows: KHCO_3_ > NaHCO_3_ > K_2_SO_4_ (Figures 4e), and the |Z| values of B‐Sn‐A in KHCO_3_, NaHCO_3_ and K_2_SO_4_ follow: KHCO_3_ < NaHCO_3_ < K_2_SO_4_, suggesting the accelerated electron transfer of e‐CO_2_RR in KHCO_3_ further (Figure 4e; Figure S24, Supporting Information).^[^ ^26^, ^29^ ^]^

**Figure 5:**
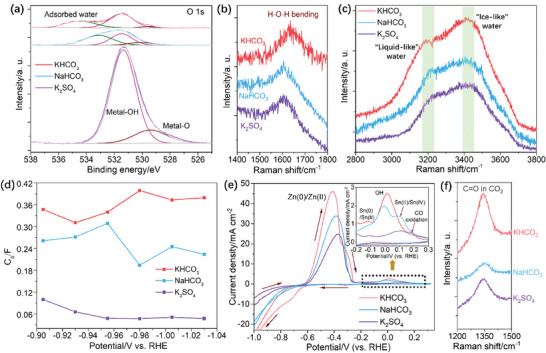
a) XPS O 1s spectra. In situ Raman spectra of b) 1400–1800 and c) 2800–3800 cm^−1^. d) Plots of C_φ_‐potential, e) CV curves in the potential range of −1.0–0.3 V and (Inset) −0.2–0.3 V, f) In situ Raman spectra of 1200–1400 cm^−1^.

It has been known that the balance between the water dissociation and hydrogen evolution reaction (HER) prevention is critical in e‐CO_2_RR.^[^ ^30^ ^]^ Therefore, the states of adsorbed water and H (or proton) in e‐CO_2_RR should warrant systematically investigations. First, in KHCO_3_ without CO_2_, B‐Sn‐A shows the smallest Tafel slopes among the three electrolytes (Figure S20, Supporting Information), suggesting the water molecule cleavage in KHCO_3_ is more beneficial.^[^ ^31^ ^]^ Meanwhile, the XPS O 1s spectra show that the intensity of water chemical adsorption peak of B‐Sn‐A in KHCO_3_ is higher, and the location is more positive than those of NaHCO_3_ and K_2_SO_4_, indicating the chemical water adsorption in KHCO_3_ is more beneficial (**Figure** **5**a). Notably, in situ Raman spectra show that the characteristic peak of H‐O‐H bending mode (d_H‐O‐H_, ≈1600 cm^−1^) of the interfacial water on the B‐Sn‐A in KHCO_3_ is blue‐shifted than those in NaHCO_3_ and K_2_SO_4_, suggesting it has less mobility and highly restriction (Figure 5b).^[^ ^32^ ^]^ However, in KHCO_3_, the OH oscillation peaks of tetrahedrally coordinated H‐bonded water (“liquid‐like water”, ≈3200 cm^−1^) and the trihedrally coordinated H‐bonded water (“ice‐like” water, ≈3400 cm^−1^) show red‐shift compared with those in NaHCO_3_ and K_2_SO_4_, suggesting the hydrogen bond networks are looser (Figure 5c).^[^ ^33^ ^]^ Last but not the least, the B‐Sn‐A demonstrates larger pseudo capacitance of surface hydrogen adsorption (C_φ_) in KHCO_3_ than those in NaHCO_3_ and K_2_SO_4_ consistently, implying that the proton concentration and coverage are enhanced through water dissociation promotion and competitive HER prevention (Figure 5d; Table S2, Supporting Information).^[^ ^34^ ^]^ All of these results confirming the reconstructed structure of B‐Sn‐A in KHCO_3_ can both promote the proton accumulation by enhancing the water dissociation (H_2_O + e^−^ → H + OH^−^) and suppress the competitive HER by suppressing the mobility of interfacial water.

To explore the oxidation states and intermediates on the surface further, we conducted a CV experiment scanning from negative to positive potential after the 3‐h tests in KHCO_3_, NaHCO_3_ and K_2_SO_4_. For all the samples, the peaks at ≈−0.4, −0.3, −0.15, 0.0, and +0.1 V are be attributed to the Zn^0^‐Zn^2+^ conversion,^[^ ^35^ ^]^ HCO_3_^−^ desorption,^[^ ^36^ ^]^ Sn^0^/Sn^2+^ conversion, OH^−^ desorption,^[^ ^37^ ^]^ and Sn^2+^/Sn^4+^ conversion,^[^ ^6^ ^]^ respectively. The intensities of Zn and Sn oxidation peaks of KHCO_3_ are higher than those of NaHCO_3_ and K_2_SO_4_, indicating the amount of exposed Zn and Sn sites are larger, which should be responsible for its high e‐CO_2_RR performance and corroborate the C_dl_ result. The peak current density of OH^−^ desorption in KHCO_3_ is larger than that of NaHCO_3_ and K_2_SO_4_, suggesting the surface enrichment of OH^−^ in KHCO_3_ for hampering the Heyrovsky step (Figure 5e).^[^ ^38^ ^]^ Notably, the oxidation peak at ≈+0.2 V represents the adsorbed CO oxidation, which can be used to evaluate the CO affinity.^[^ ^39^ ^]^ We can see that the B‐Sn‐A in KHCO_3_ and NaHCO_3_ show negligible adsorbed CO oxidation peak than that in K_2_SO_4_ (Inset in Figure 5e), underling the CO_2_‐to‐CO competitive reaction is suppressed in bicarbonate electrolytes. Additionally, the in situ Raman spectra measured at 1200–1400 cm^−1^ after test show that the intensity of the C═O vibrations peak of CO_2_ in KHCO_3_ is stronger than those in NaHCO_3_ and K_2_SO_4_, implying the reconstructed Sn‐SnO_2_/ZnO in KHCO_3_ has stronger CO_2_ affinity,^[^ ^40^ ^]^ which should be responsible for CO_2_‐to‐HCOO^−^ conversion (Figure 5f).

Combine with the structure and mechanism studies, we believe that the reconstructed Sn‐SnO_2_/ZnO in KHCO_3_ has high effective surface area that enhances the CO_2_ adsorption. Moreover, the positively charged Sn and Zn sites on surface shall benefit for the OH^−^ adsorption and promoting the O‐down configuration of water molecules adsorption, that benefit for the water cleavage and suppress the Heyrovsky step of HER.^[^ ^41^ ^]^ Also, the *OCO adsorption configuration of CO_2_ (O atom adsorption configuration) shall be favorable on the positively charged Sn and Zn sites.^[^ ^42^ ^]^ All these shall be the issues of e‐CO_2_RR enhancement of B‐Sn‐A in KHCO_3_.

## 3 Conclusion

In summary, we report a novel finding that the surface reconstruction of electrocatalyst in the e‐CO_2_RR process strongly depends on the electrolyte used, which is responsible for the efficiency of electrochemical CO_2_‐to‐fuel conversion. The surface of B‐Sn‐A reconstructs in the e‐CO_2_RR process through element leaching‐redeposition and proceed the Sn‐SnO_2_/ZnO, the ZnO encapsulated Sn‐SnO_2_/ZnO, and the Sn‐SnO_2_/Zn‐ZnO in KHCO_3_, NaHCO_3_ and K_2_SO_4_, respectively. Typically, the Sn‐SnO_2_/ZnO reconstructed in KHCO_3_ achieves high CO_2_‐to‐HCOO^−^ conversion performance (highest FE HCOO^−^ ≈89%, yield rate ≈0.58 mmol cm^−2^ h^−1^ and the j_HCOO‐_ ≈31.14 mA cm^−2^) at −0.93 V. Most importantly, we find that the reconstructed Sn‐SnO_2_/ZnO of B‐Sn‐A in KHCO_3_ has increased positive charge on the active sites Zn and Sn, which account for superior CO_2_‐to‐HCOO^−^ conversion by: i) accelerating the charge/electron transportation; ii) improving the CO_2_ adsorption and optimizing the CO_2_ adsorption configuration; iii) promoting the proton concentration through enhancing the water adsorption/cleavage and limiting the HER. Our findings may provide insightful understanding on the mechanism of electrolyte‐dependent catalytic performance and presents efficient approaches for guiding the design of novel electrocatalyst and choice of electrolyte for effective e‐CO_2_RR.

## 4 Experimental Section

### Chemicals and Reagents

Tin (II) sulfate (SnSO_4_·6H_2_O, >99%), sodium hydroxide (NaOH, >97%), hydrochloric acid (HCl, ∼36%), potassium persulfate (K_2_S_2_O_8_, >99%), potassium bicarbonate (KHCO_3_, >99%), and ethanol were purchased from Aladdin, Co. Ltd. Brass plate (H62, Cu weight content = 62%, Zn = residual) was purchased from Wanda Scientific Materials, Xingtai, Hebei. All chemicals were utilized as received without any treatment. Deionized (DI) water was supplied by a Barnstead Nanopure water system (resistivity: 18.3 MΩ cm^−1^) and was used for the preparation of all aqueous solutions.

### Electrocatalyst Fabrication

0.1 g SnSO_4_ and 0.1 g K_2_S_2_O_8_ were added in 15 mL of 1 m NaOH with vigorous stirring until the solution become clear. Then a piece of brass plate (B) (4 cm × 2 cm, thickness = 0.1 mm) after cleaning (first polished by 200‐grid abrasive paper then soaked in 1 m NaOH solution for 20 min, and finally washed by DI water) was used as cathode, a piece of commercial Ti mesh with Ru‐Ir oxide coating was used as anode, and the deposition time is 15 min under with the current density of 2.5 mA cm^−2^. After that, the brass plate with electrodeposited Sn (B‐Sn) was placed in a parcel boat and annealed in air for 6 h at 500 °C by using a tube‐oven (Ke Jing Co. Ltd. Hefei, OTF‐1200X). The B, the brass plate after annealing in air at 500 °C (B‐A) and the B‐Sn were used as comparisons.

### Materials Characterizations

The crystal structures characterizations were conducted on a powder X‐ray diffraction (XRD) (Rigaku rotating anode diffractometer with a mono chromated Cu Kα X‐ray source). The morphology and chemical composition were determined by scanning electron microscope (SEM) (ZEISS‐Merlin) and transmission electron microscope (TEM) (JEM‐F200) coupled with energy dispersive spectrometer (EDS). The cross‐section TEM sample was obtained by using focused ion beam (FIB) technique. Raman spectra were recorded by a confocal micro‐Raman system (Horiba, LABHRev‐UV) using 532 nm laser as excitation wavelength, with an acquisition time of 10 s. In situ Raman spectrum was measured by using a Teflon in situ Raman cell, with 1 mm thickness of 0.5 m KHCO_3_ saturated by CO_2_ as electrolyte. The Raman excitation wavelength is 532 nm, with 100% power ratio and the acquisition time of 20 s. The current intensities were directly controlled by an electrochemical workstation (CHI 760E). X‐ray photoelectron spectroscopy (XPS) was measured on a Thermo Fisher Scientific Theta Probe with Mg Ka (hν = 1253.6 eV) as the excitation source. The Ar^+^ ion sputtering method was held with etching rate of 0.52 nm s^−1^ (taking Ta_2_O_5_ etching rate as reference), and the spectrum was collected every 15 s. The electronic spin resonance (ESR) spectra were measured on an electron spin/paramagnetic resonance spectrometer (Magnettech, MS‐5000). Quasi in situ ESR measurements were carried out at 5th min after the 3‐h test. Element concentration was measured on an inductively coupled plasma mass spectrometry (ICP‐MS, Agilent) using 5% HNO_3_ as digestive reagent.

### Electrochemical Test

The electrochemical experiments were performed in an H‐type cell separated by a Celgard 2500 membrane through a conventional three‐electrode system linked with an electrochemical workstation (CHI 660E). The 0.5 m KHCO_3_, 0.5 m NaHCO_3_, and 0.25 m K_2_SO_4_ saturated by CO_2_ were used as electrolytes, respectively. The as‐prepared samples were directly used as the working electrodes, a Ti mesh with Ru‐Ir oxide coating (Ru‐Ir‐Ti mesh) was utilized as the counter electrode, and a Hg/Hg_2_Cl_2_ in saturated KCl was used as the reference electrode. All the data reported without iR‐compensation. Prior to the CO_2_ reduction, the electrolyte was plugged by CO_2_ for at least 15 min to remove the residual air and have a fully CO_2_‐saturated solution. The galvanostatic tests were held at different potentials for 3 h. All the potentials were converted to the reversible hydrogen electrode (RHE) via the Nernst equation (E vs RHE = E vs Hg/Hg_2_Cl_2_ + 0.0591 × pH + 0.241 V). The electronic impedance spectroscopy (EIS) plots were measured in the frequency 0.01–10^6^ Hz with an amplitude of 10 mV. The EIS raw data were fitted by ZView. The flow cell measurement was carried out in a homemade 2‐electrode cell with a geometric area of 2 × 2 cm. The cathode catalyst was B‐Sn‐A, a Celgard 2500 membrane and a Ti mesh (thickness = 0.2 mm) was used as membrane and anodic catalyst, respectively. The catholyte was CO_2_‐saturated 0.5 m KHCO_3_, and the anolyte was 0.5 m KHCO_3_, circulate in the groove of the cathode and anode plates through a peristaltic pump at a speed of 50 mL min^−1^, respectively.

### Products Analysis

The qualitative analysis of formate was held on ultraviolet‐visible spectroscopy (Shimadzu UV‐2600) (Shimadzu, Kyoto, Japan). The detection wavelengths were from 185 to 400 nm. The KHCO_3_ and NaHCO_3_ electrolytes before and after tests were mix with 1.0 m HCl with volume ratio of 1:1. The electrolytes before test after HCl mixing were used as references. The K_2_SO_4_ electrolyte after tests were measured directly, and the K_2_SO_4_ before test was used as reference. The liquid products were analyzed using high‐performance liquid chromatography (HPLC, Agilent 1260) with a refractive index detector (RID). The flow phase was de‐ionized water, and the flow rate of 0.6 mL min^−1^. The solution volume of each injection batch was 15 µL. The carbon‐based gaseous products were measured by gas chromatography (GC) (7890B, Agilent Technologies) with a flame‐ionized detector (FID), and the hydrogen gas was measured by GC with a thermal conductivity detector (TCD).

The FEs of the products were calculated as the Equation (1):

(1) $$FE=\frac{n}{n_{0}}$$

*n* represents the amount of product, mol; *n_0_* represents the theoretical yield of product, mol.

The *n_0_* can be calculated as Equation (2):

(2) $$n_{0}=\frac{Q}{zF}$$

*Q* represents the total charge consumption of the electrochemical process, C; *z* represents the number of electron transfer. For formate, CO and H_2_, *z* = 2; for CH_4_, *z* = 8; for C_2_H_4_, *z* = 12. *F* represents Faraday constant, 96 485 C mol^−1^.

The partial current density of HCOO^−^ (j_HCOO‐_) (mA cm^−2^) is calculated as Equation (3):

(3) $$j_{\mathrm{HCOO}-}=\frac{Q}{t}\times FE$$

The t represents the reaction time, s.

The electrochemical surface area (ECSA) of as‐prepared sample was estimated from their double layer capacitance (C_dl_), which has been measured using simple cyclic voltammetry method. Here the applied potential window was non‐Faraday region.^[^ ^43^ ^]^ Then the current was only generated for charging of double layer, which was expected to have linear relationship with active surface area. The calculated slope is two times of C_dl_. The value of ECSA can be calculated according to the Equation (4):

(4) $$ECSA=C_{dl}/C_{s}$$

The specific capacitance value of 𝑪_𝐬_ is 0.040 mF cm^−2^.^[^ ^44^ ^]^

## Conflict of Interest

The authors declare no conflict of interest.

## Supporting information

Supporting Information

## Data Availability

The data that support the findings of this study are available from the corresponding author upon reasonable request.
