# Supplementary material for: Electrolyte‐Assisted Structure Reconstruction Optimization of Sn‐Zn Hybrid Oxide Boosts the Electrochemical CO2‐to‐HCOO− Conversion
Source: Adv Sci (Weinh). 2024 Aug 19;11(39):2407019. doi: 10.1002/advs.202407019 (PMC11497031; doi:10.1002/advs.202407019)
Supplement: Supplementary file 1 — Supporting Information [file ADVS-11-2407019-s001.docx]

**Supporting Information**

**Electrolyte-assisted Structure Reconstruction Optimization of Sn-Zn Hybrid Oxide Boosts the Electrochemical CO_2_-to-HCOO^-^ Conversion**

Jinxian Feng ^a^, Chunfa Liu ^a^, Lulu Qiao ^a^, Keyu An ^a^, Sen Lin ^c^, Weng Fai Ip ^b^, and Hui Pan^* a, b^

*^a^ Institute of Applied Physics and Materials Engineering, University of Macau, Macao S. A. R., 999078, China*

*^b^ Department of Physics and Chemistry, Faculty of Science and Technology, University of Macau, Macao S. A. R., 999078, China*

*^c^ State Key Laboratory of Photocatalysis on Energy and Environment, College of Chemistry, Fuzhou University, Fuzhou, 350108, China*

*Corresponding author: Hui Pan, huipan@um.edu.mo (email), +853-88224427 (tel.), +853-88222454 (fax).


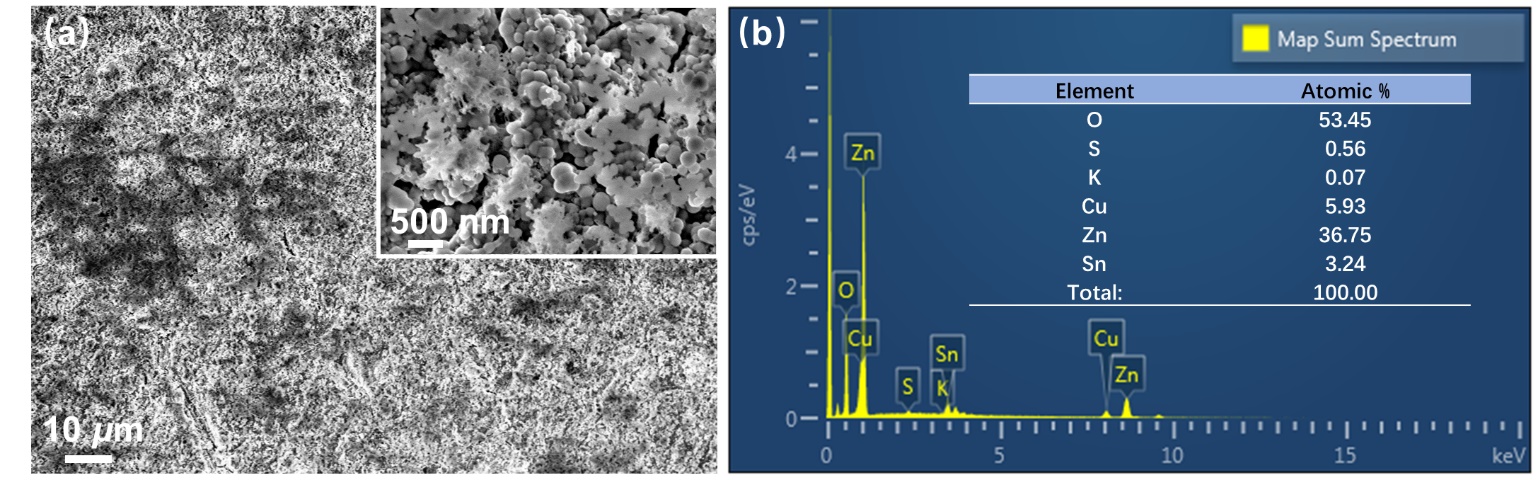


**Figure S1.** (a) SEM images and (b) EDS spectrum of B-Sn-A pre-electrocatalyst.

**Figure S2.** XRD pattern of B-Sn-A pre-electrocatalyst.

The XRD pattern of B-Sn-A pre-electrocatalyst shows the diffraction peaks of brass (JCPDS 50-1333), bronze (JCPDS 45-1488) and Sn (JCPDS 04-0672), which are coming from substrate. And the diffraction peaks of ZnO (JCPDS 36-1451) and SnO_2_ (JCPDS 41-1445) can also be seen, which should be attributed to oxidized Zn and oxidized Sn, respectively.

**Figure S3.** Raman spectrum of B-Sn-A pre-electrocatalyst.


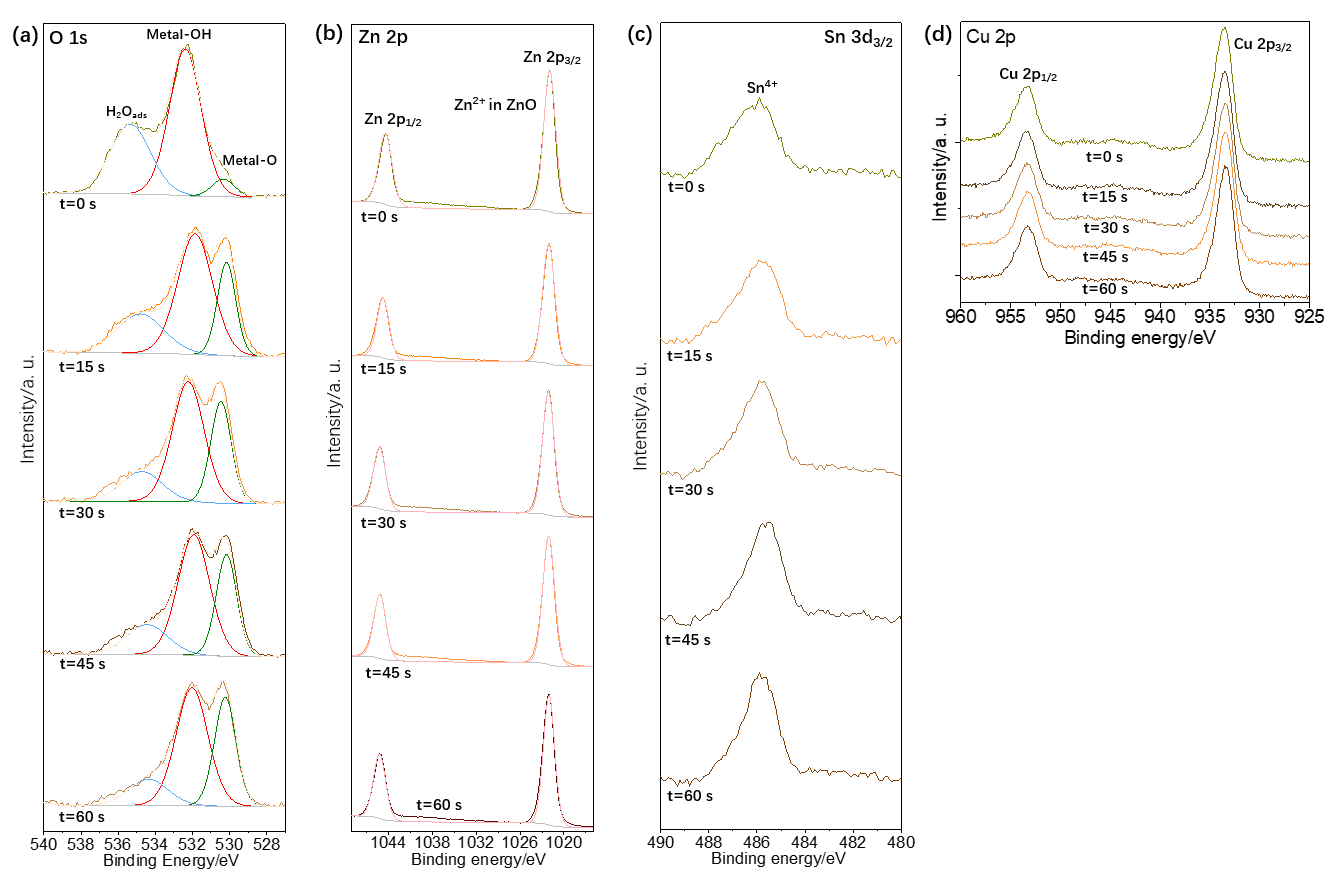


**Figure S4.** XPS spectra of B-Sn-A pre-electrocatalyst by Ar^+^ sputtering: (a) O 1s, (b) Sn 3d_5/2_, (c) Zn 2p and (d) Cu 2p.

The XPS O 1s spectra of B-Sn-A pre-electrocatalyst show the signals for metal-O and metal-OH (Figure S4a),^[1]^ suggesting the existence of metal oxide. Meanwhile, the XPS Zn 2p, Cu 2p and Sn 3d_3/2_ spectra show that Zn, Cu and Sn are assigned to Zn^2+^ of Zn oxide,^[2, 3]^ Cu^2+^ of Cu oxide,^[4]^ and Sn^4+^ of Sn oxide,^[5]^ respectively (Figure S4b-d).

The XRD, Raman and XPS results show that Cu in brass is alloyed with Sn partially, resulting in bronze. Meanwhile, Zn and Cu diffuse onto the surface and are oxidized in the annealing process.


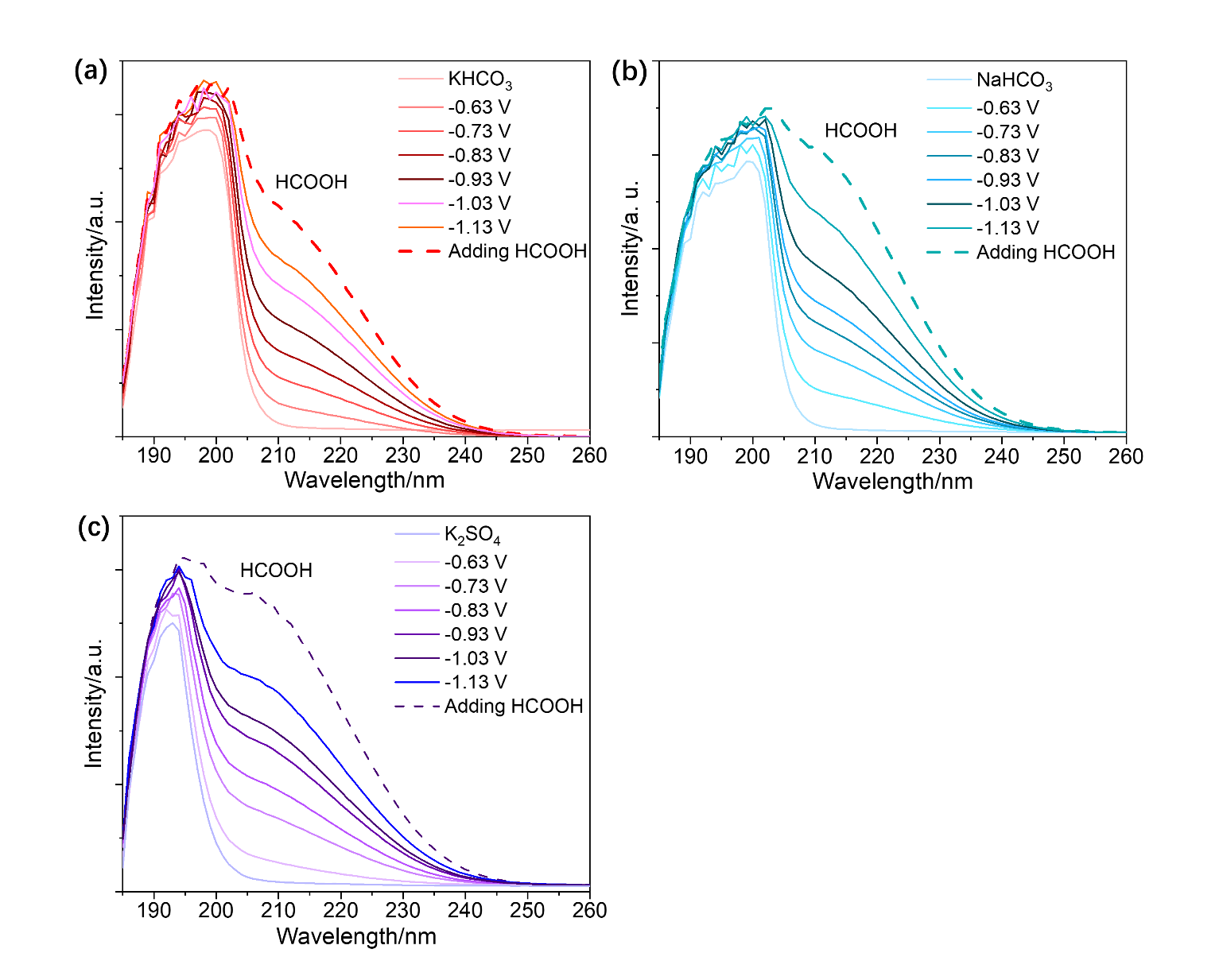


**Figure S5.** UV-vis spectra for B-Sn-A tested in (a) 0.5 M KHCO_3_, (b) 0.5 M NaHCO_3_ and (c) 0.25 M K_2_SO_4_ (Dash lines: reference + HCOOH).

The electrolytes after tests were treated as the method describes in the experimental section. Comparing with the electrolytes before tests, the peaks locate at wavelength of ~210 nm presence after 3-h e-CO_2_RR tests, and the peak heights increase with the HCOOH adding, indicating the existence of HCOO^-^.


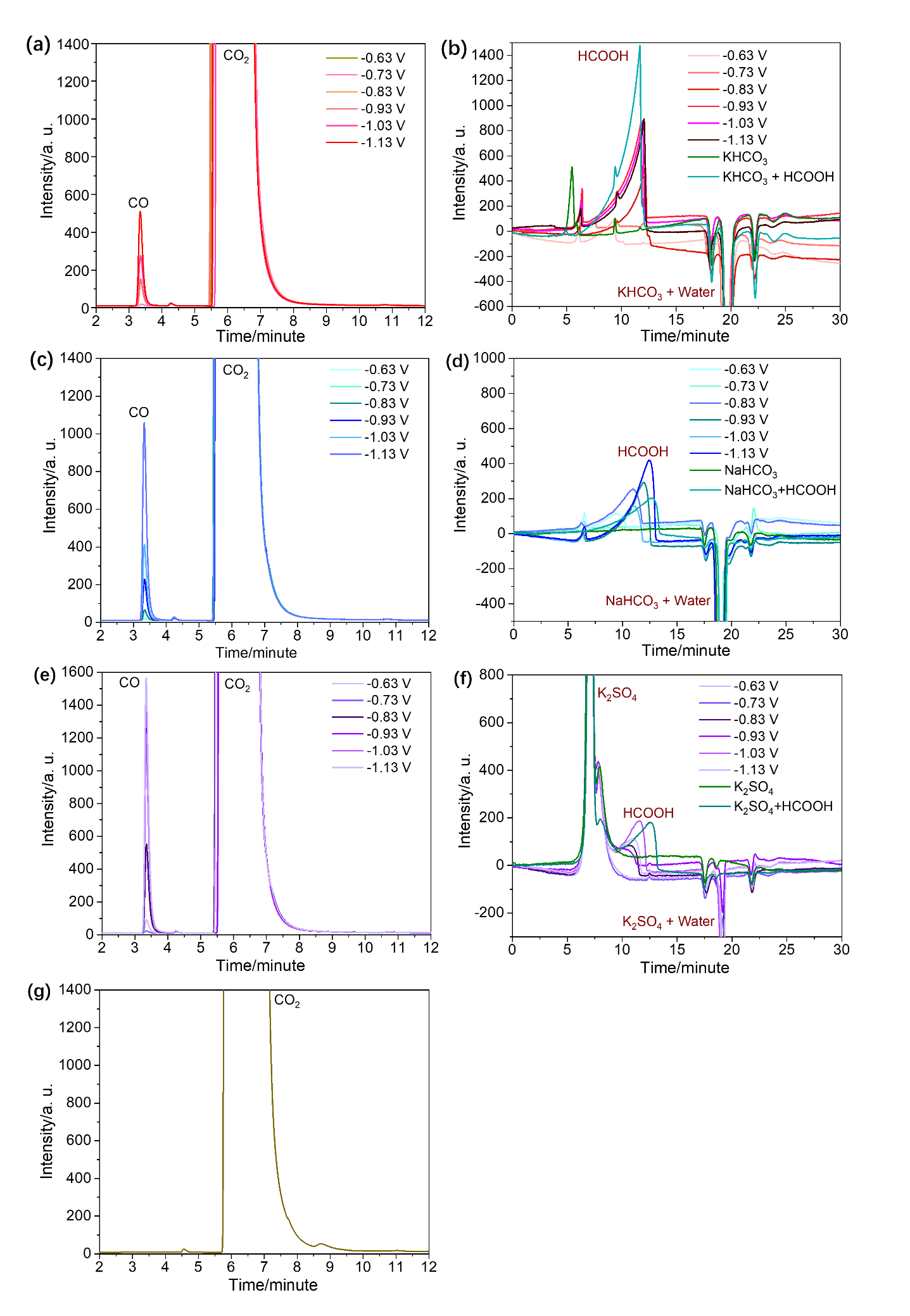


**Figure S6.** GC (a, c, e) and HPLC chromatograms (b, d, f) of B-Sn-A measured at different potentials using KHCO_3_ (a-b), NaHCO_3_ (c-d) and K_2_SO_4_ (e-f). (g) CG chromatogram of used CO_2_ gas.


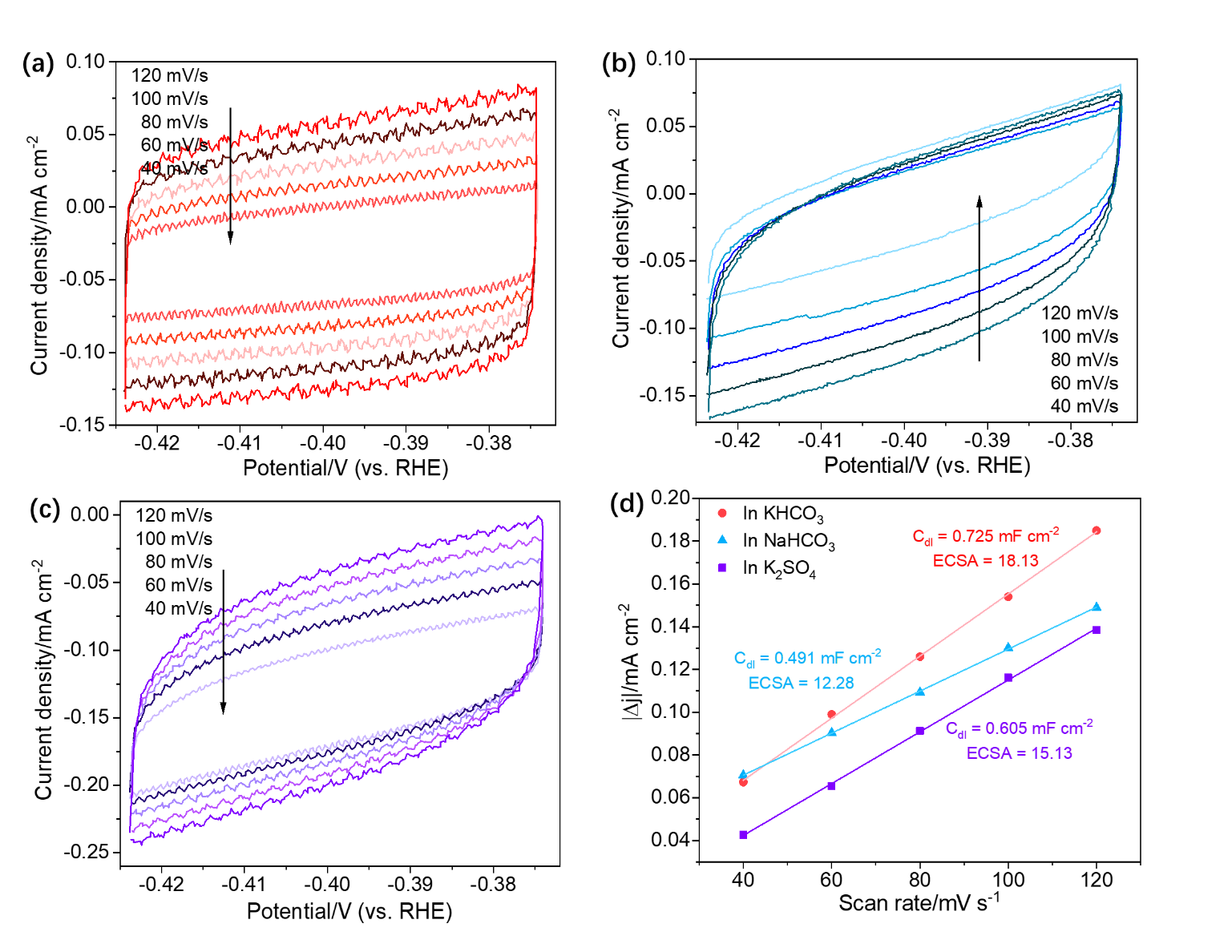


**Figure S7**. CV curves at different scan rates for B-Sn-A in (a) 0.5 M KHCO_3_, (b) 0.5 M NaHCO_3_ and (c) K_2_SO_4_. (d) double-layer capacitance (C_dl_) measurements and ECSAs of B-Sn-A in different electrolytes.

**Figure S8.** j_HCOO-_ of B-Sn-A in different electrolytes calculated based on ECSA.


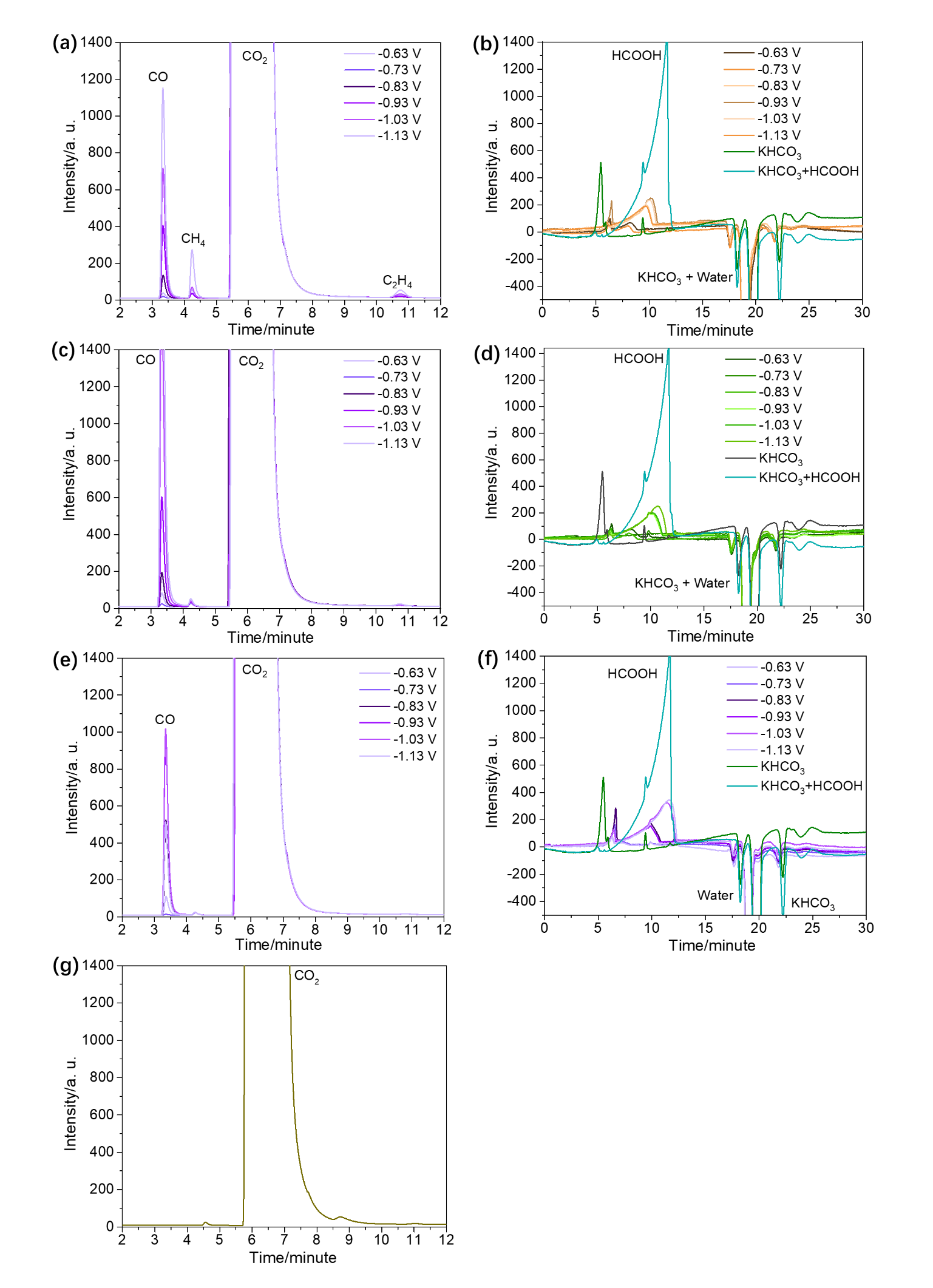


**Figure S9.** GC (a, c, e) and HPLC chromatograms (b, d, f) of B, B-A and B-Sn measured at different potentials. (g) CG chromatogram of used CO_2_ gas.


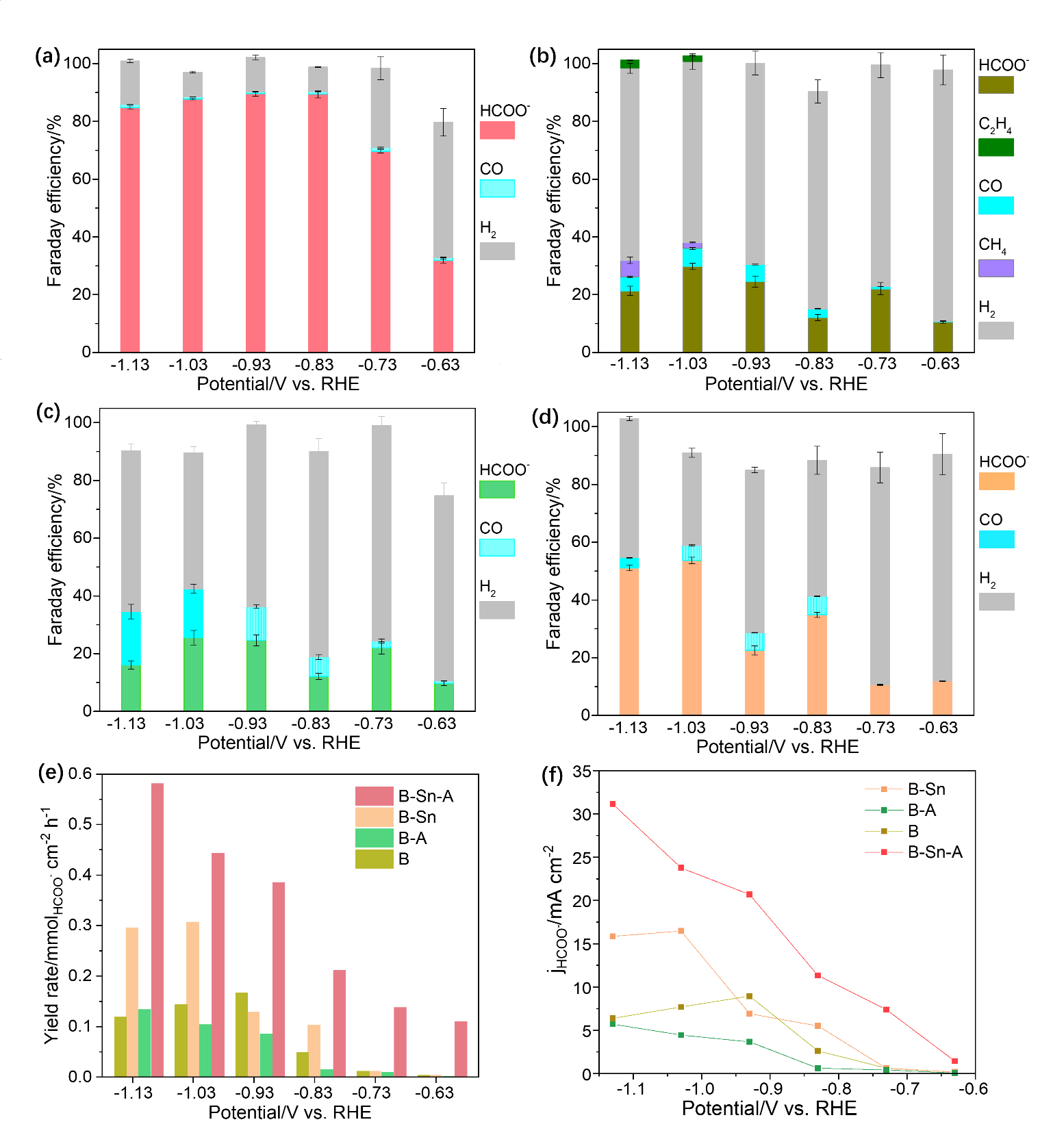


**Figure S10.** Distribution of products: (a) B-Sn-A, (b) B, (c) B-A and (d) B-Sn. (e) j_HCOO-_ and (f) HCOO^-^ yield rates.

The gas products of B are CO, CH_4_, C_2_H_4_ and H_2_, while those of B after annealing in air at 500℃ (B-A), brass with Sn electrodeposited (B-Sn) and B-Sn-A are CO and H_2_ only (Figures S10a-d). The HCOO^-^ is the only liquid product for all the samples (Figures S10a-d). Typically, B-Sn-A has a HCOO^-^ FE of > 85% at a volage range of -0.83 ~ -1.13 V, where the highest HCOO^-^ FE (~ 89%) occurs at -0.93 V (Figure S10a), and also shows a high j_HCOO-_ and yield rate (20.68 mA cm^-2^ and 0.38 mmol h^-1^ cm^-2^ at -0.93 V, and 31.14 mA cm^-2^ and 0.58 mmol h^-1^ cm^-2^ at -1.13 V, respectively) (Figure S10e-f). On the contrary, B only shows the highest HCOO^-^ FE of ~32 % at -0.93 V (Figure S10b) and has the highest j_HCOO-_ (8.96 mA cm^-2^) and yield rate (0.17 mmol h^-1^ cm^-2^) at -1.03 V (Figure S10e-f). B-A shows the highest HCOO^-^ FE of 35% at -1.03 V (Figure S10c), and has the highest j_HCOO-_ of 5.72 mA cm^-2^ and HCOO^-^ yield rate of 0.13 mmol h^-1^ cm^-2^ at -1.13 V (Figure S7e-f). The B-Sn shows the highest HCOO^-^ FE (35%) (Figure S7d), j_HCOO-_ (16.46 mA cm^-2^) and HCOO^-^ yield rate (0.31 mmol h^-1^ cm^-2^) at -1.03 V (Figure S10e-f).

**
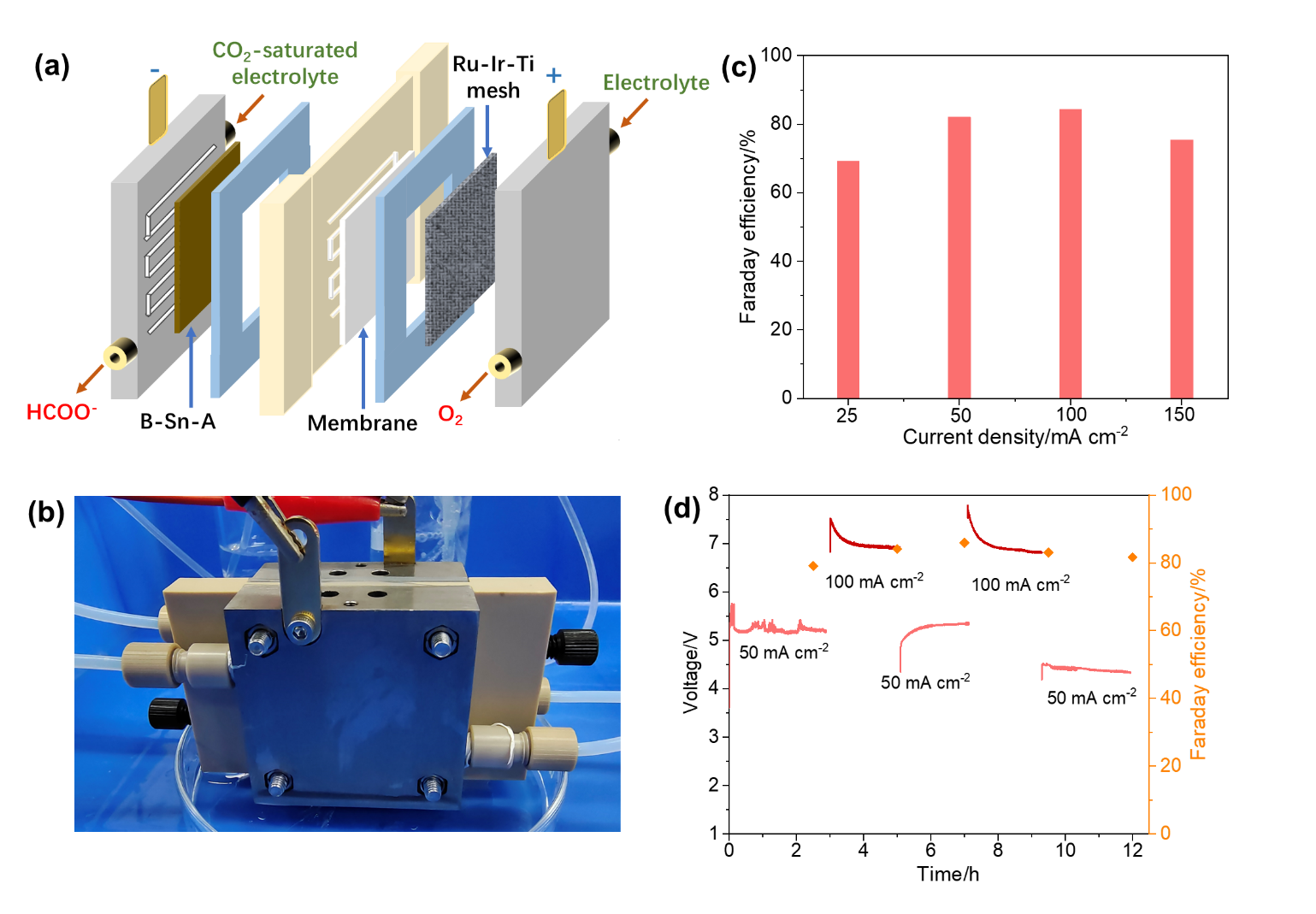
**

**Figure S11.** (a) Scheme of flow cell assembly. (b) Photo of 2-electrode flow cell. (c) HCOO^-^ FEs measured at different current densities. (d) On-and-off measurements at different current densities.

**Table S1.** Comparison of the B-Sn-A electrocatalyst to some other e-CO_2_RR electrocatalysts for HCOO^-^ generation with similar compositions.

| **Electrocatalyst** | **Electrolyte** | **FE HCOO^‑^**  **(Potential/V vs. RHE)** | **HCOO^‑^ yield rate/ mmol cm^-2^ h^-1^ (Potential/V vs. RHE)** | **Reference** |
| --- | --- | --- | --- | --- |
| B-Sn-A | 0.5 M KHCO_3_ | 31.89 (-0.63)  69.67 (-0.73)  89.39 (-0.83)  89.60 (-0.93)  87.71 (-1.03)  84.82 (-1.13) | 0.110 (-0.63)  0.137 (-0.73)  0.211 (-0.83)  0.385 (-0.93)  0.443 (-1.03)  0.580 (-1.13) | Our work |
| SnPS_3_ | 0.5 M KHCO_3_ | 22.3 (-0.65)  14.7 (-0.75) | 0.090 (-0.65)  0.133 (-0.75) | *Angew. Chem. Int. Ed.*, 2023, 62, e202217253 |
| SnO_2_/ZnO composite HNF | 0.5 M KHCO_3_ | 50 (-1.19) | 0.037 (-1.19) | *ACS Sus. Chem. Eng.*, 2020, 8, 29, 10639. |
| np-Cu11Sn1 | 0.5 M KHCO_3_ | 68 (-0.9)  72.1 (-1.0)  69 (-1.1) | 0.140 (-0.9)  0.203 (-1.0)  0.280 (-1.1) | *Chem. Commun*., 2023, DOI: 10.1039/D3CC04825J |
| SnS_2_ nanoplates | 0.5 M KHCO_3_ | 78 (-0.8)  80 (-0.9)  82 (-1.0)  86 (-1.1) | 0.019 (-0.8)  0.222 (-0.9)  0.334 (-1.0)  0.426 (-1.1) | *Appl Catal B: Environ.*, 2024, 341, 123274. |
| Cu/Ti_3_C_2_T_x_ | 0.1 M NaHCO_3_ | 58.6 (-0.73) | 0.012 (-0.73) | *J. Mater. Chem. A.*, 2022, 10, 1965. |
| Pd–B/C | 0.1 M KHCO_3_ | 70 (-0.5) | 0.131 (-0.5) | *J. Am. Chem. Soc.*,  2018, 140, 2880 |
| Zn-Vo-SnO_2_ | 0.5 M KHCO_3_ | 82 (-0.8)  80 (-0.9) | 0.229 (-0.8)  0.403 (-0.9) | *Mater. Today Energy.,* 2023, 35, 105819. |
| SnO/C | 0.5 M KHCO_3_ | 65 (-0.85) | 0.103 (-0.85) | *Angew. Chem. Int. Ed.*,  2018, 57, 2943 |
| CuO/SnO_2_ | 0.5 M KHCO_3_ | 54.8 (-0.85)  38 (-0.95) | 0.079 (-0.85)  0.087 (-0.95) | *Angew. Chem. Int. Ed.*,  2023, 2023,62, e2023064 |
| Zn_2_SnO_4/_ZnO | 0.5 M KHCO_3_ | 85 (-0.9)  87 (-1.0) | 0.468 (-0.9)  0.576 (-1.0) | *Inorg. Chem. Front.*, 2023, 10, 1818. |
| SnO | 0.5 M KHCO_3_ | 78 (-0.96) | 0.746 (-0.96) | *ACS Catal.*, 2021, 11, 14986. |
| Bi-TiO_2_-700 | 0.1 M KHCO_3_ | 94 (-0.8)  95 (-1.0)  92 (-1.2) | 0.067 (-0.8)  0.139 (-1.0)  0.250 (-1.2) | *J. Am. Chem. Soc.*, 2023, 145, 25, 14133. |
| SrSnO_3_ perovskite nanowires | 0.5 M NaHCO_3_ | 70 (-1.00)  75 (-1.10) | 0.170 (-1.00)  0.238 (-1.10) | *Nano Energy.*, 2019, 62, 861 |
| CoP_2_O_6_/HCS-Cu | 0.5 M KHCO_3_ | 72.5 (-0.76) | 0.473 (-0.76) | *ACS Nano.*, 2023, 10.1021/acsnano.2c12426 |
| Sn_1–x_In_x_@In_1–y_Sn_y_O_z_ | 0.1 M KHCO_3_ | 70 (-0.9)  80 (-1.0)  75 (-1.1) | 0.056 (-0.9)  0.112 (-1.0)  0.215 (-1.1) | *Adv. Funct. Mater*. 2021, 31, 2103601 |
| Sn NPs | 0.5 M KHCO_3_ | 70 (-0.9)  80 (-0.95)  82 (-1.0) | 0.155 (-0.9)  0.168 (-0.95)  0.187 (-1.1) | *Chin. J. Catal.*, 43 (2022) 1473–1477 |
| SnO/NiCo_2_O_4_/CC | 0.2 M Na_2_SO_4_ | 83 (-0.9)  85 (-1.0)  91.87 (-1.1) | 0.248 (-0.9)  0.298 (-1.0)  0.359 (-1.1) | *Chem. Eng. J.*, 469 (2023) 144049 |
| Sn-Cu alloy | 0.5 M KCl | 82.3 (-1.14) | 1.208 (-1.14) | *Sci. Bull.*, 2020, 65, 1547. |
| Sn/SnO_x_ | 0.1 M KHCO_3_ | 68.7 (-1.13) | 0.081 (-1.13) | *Mater. Today Commun.,* 2023, 35, 105819. |
| Cu_6_Sn_5_/oxides | 0.5 M KHCO_3_ | 65 (-0.85)  90.1 (-0.95) | 0.182 (-0.85)  0.423 (-0.95) | *Adv. Energy Mater.,* 2023, 2203506 |
| SnO_2_ with surface -OH | 0.5 M KHCO_3_ | 82 (-1.0)  86 (-1.1) | 0.230 (-1.0)  0.321 (-1.1) | *Nano Energy* 2023, 108, 108193. |
| Zn_3_Sn_2_ | 0.5 M KHCO_3_ | 42 (-0.9)  78 (-1.0) | 0.118 (-0.9)  0.255 (-1.0) | *Appl. Surf. Sci.,* 2023, 108, 108193. |
| CuS/SnO_2_-S | 0.5 M KHCO_3_ | 84.9 (-0.8) | 8.86 (-0.8) | *J. Energy Chem.*  2023, 82, 497. |
| Cu_1_Sn_1_ | 0.5 M KHCO_3_ | 85 (-1.0)  87 (-1.1) | 0.119 (-1.0) | *ACS Catal.,* 2021, 11, 11103. |

**
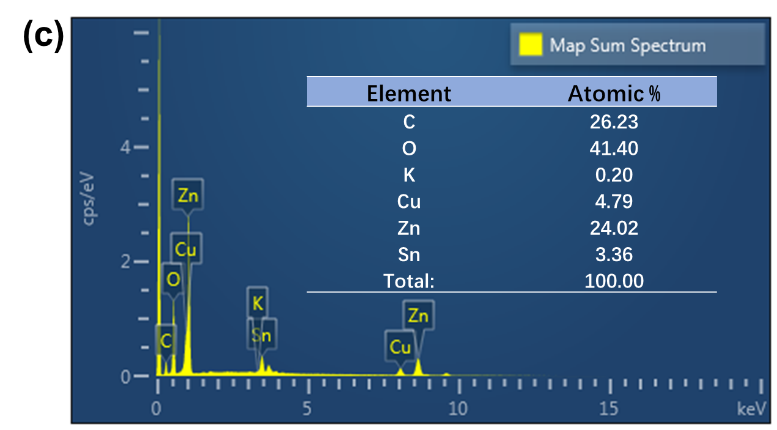
**

**Figure S12.** EDS spectrum of B-Sn-A and elemental contents after the 3-h test in 0.5 M KHCO_3_.


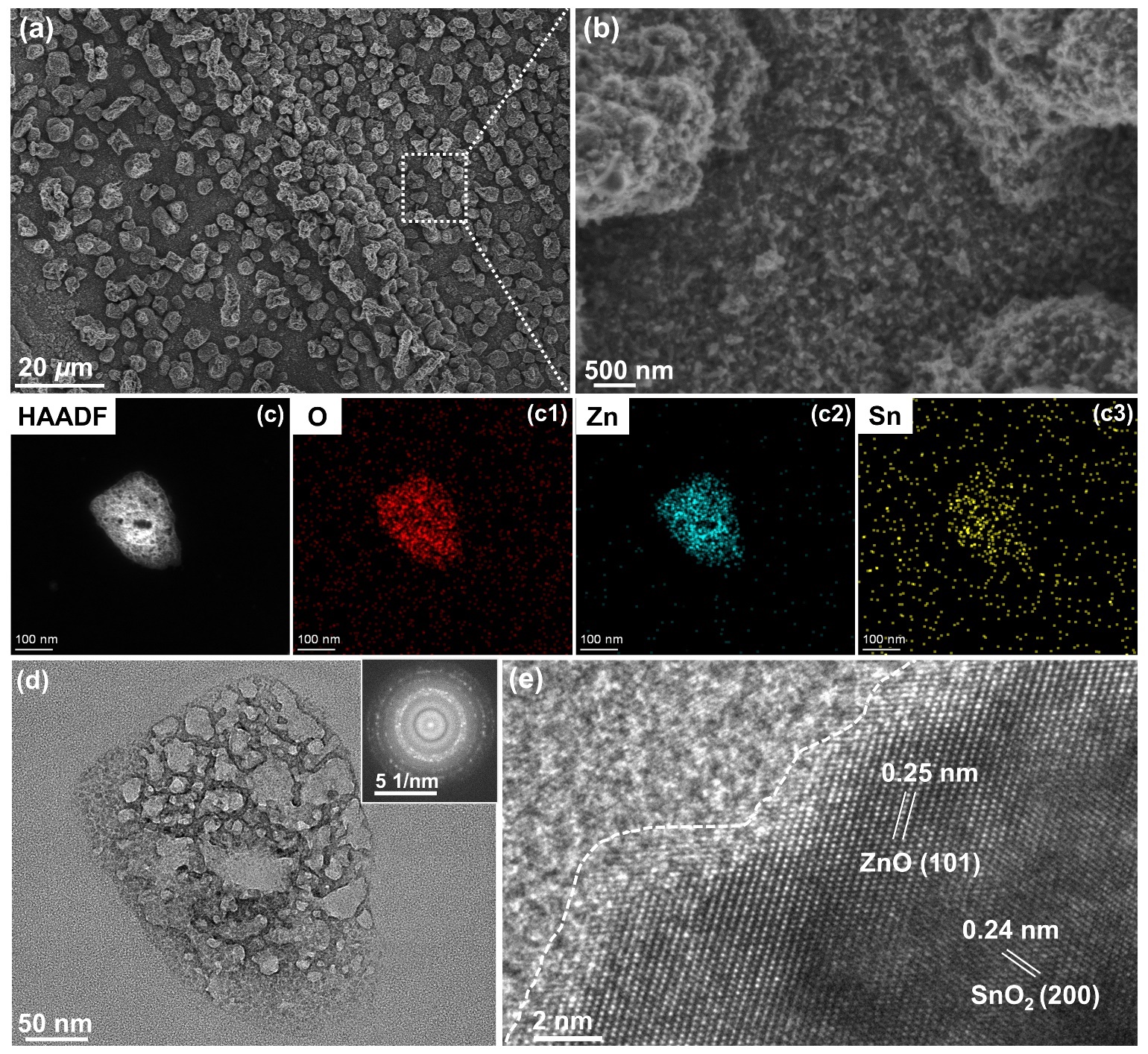


**Figure S13.** Morphology of B-Sn-A after the 3-h test in NaHCO_3_: (a-b) SEM images; (c) HAADF image; (c1-c3) EDS Zn, O and Sn mapping images; (d) TEM image (Inset in d: FFT image). (e) HRTEM images.


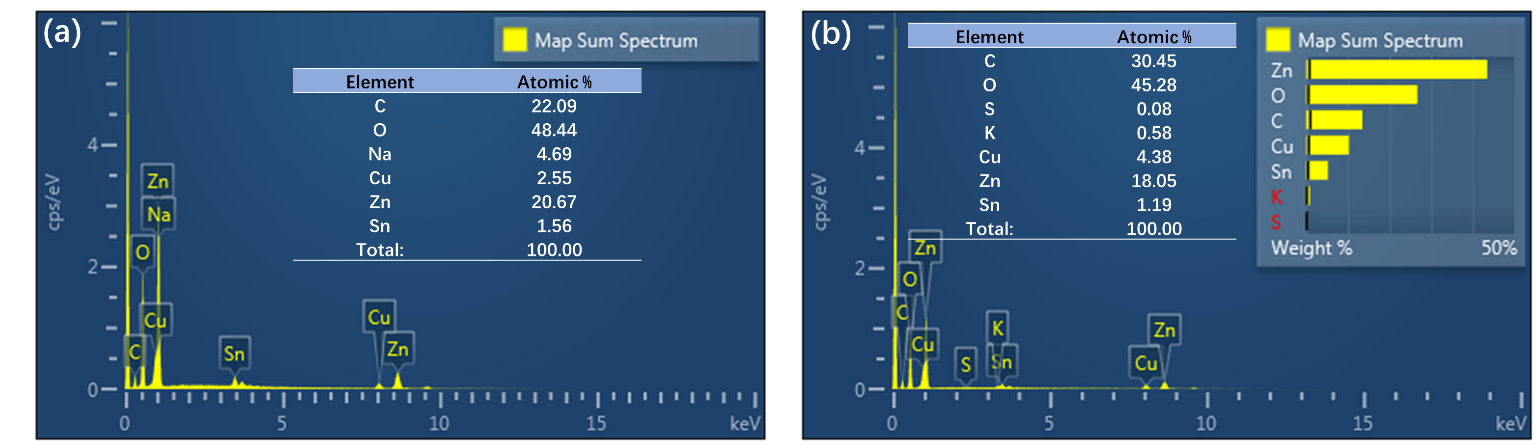


**Figure S14.** EDS spectrum of B-Sn-A and elemental contents after 3-h test in: (a) 0.5 M NaHCO_3_ and (b) 0.25 M K_2_SO_4_.


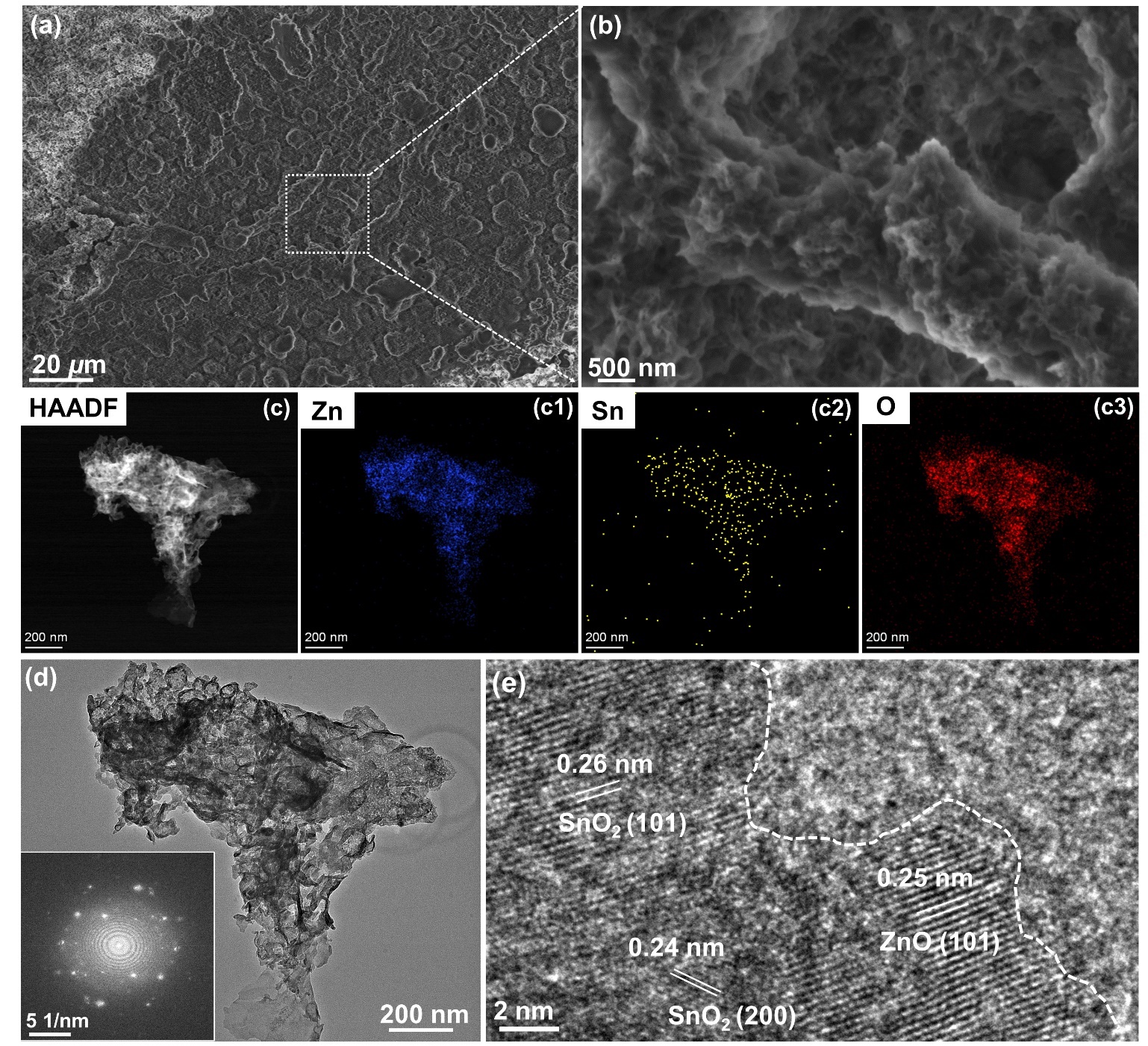


**Figure S15.** Morphology of B-Sn-A after 3-h test in K_2_SO_4_: (a-b) SEM images. (c) HAADF image and EDS mappings for Zn (c1), Sn (c2) and O (c3). (d) TEM image (Inset: FFT pattern). (e) HRTEM image.


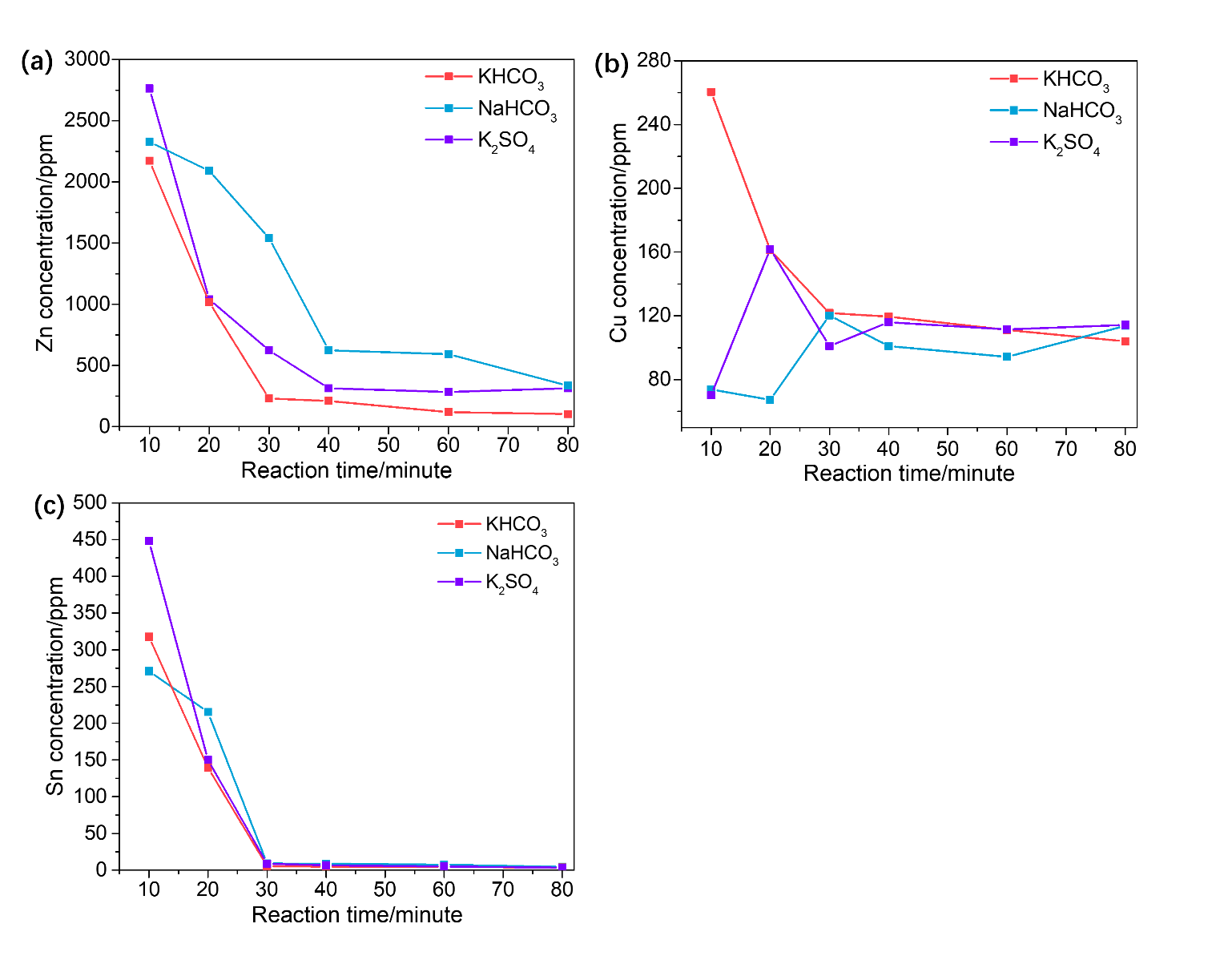


**Figure S16.** Concentrations of (a) Cu, (b) Zn and (c) Sn in different electrolytes at various reaction time.


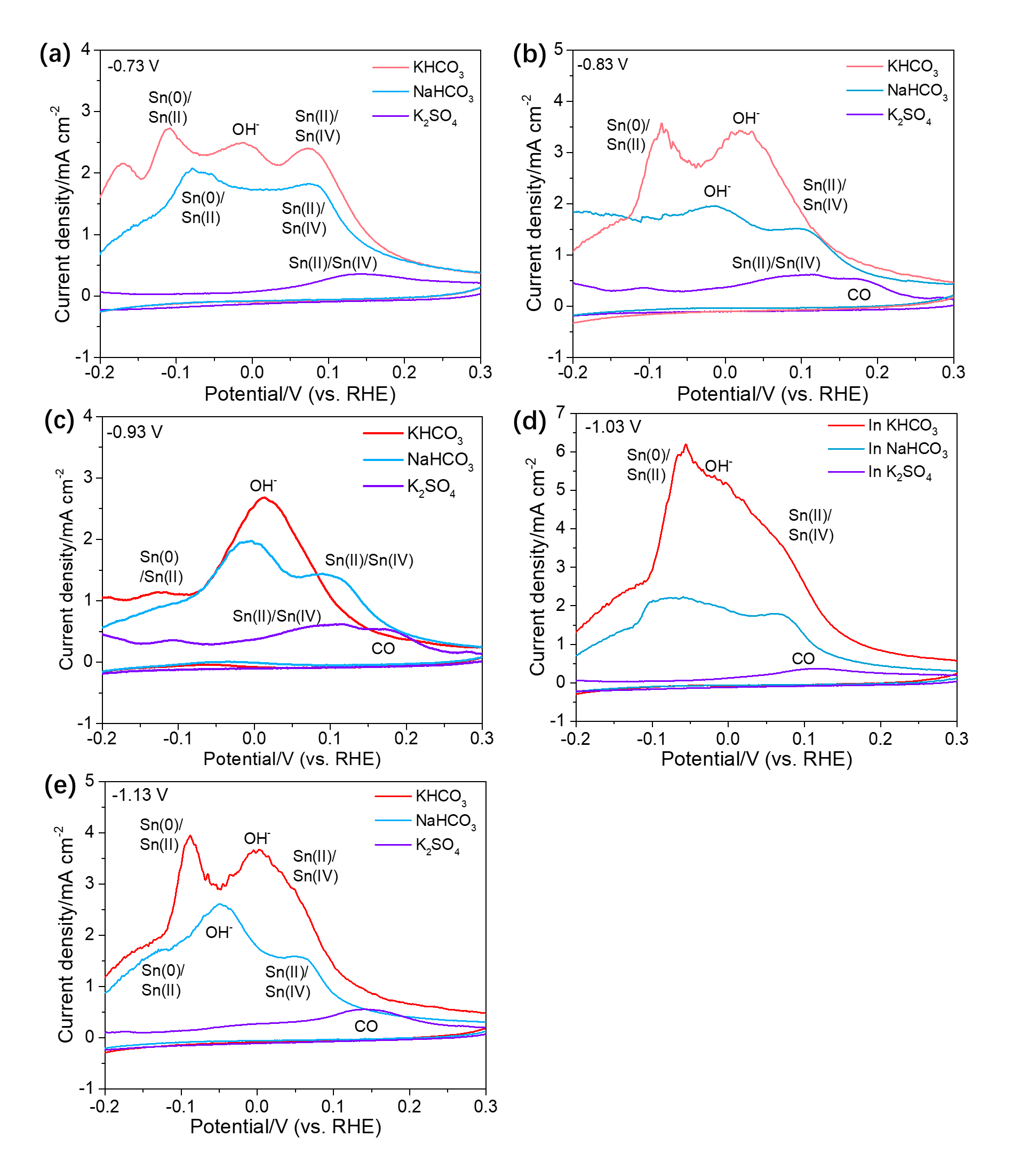


**Figure S17.** CV curves of B-Sn-A after 3-h test at (a) -0.73 V, (b) -0.83 V, (c) -0.93 V, (d) -1.03 V, (e) -1.13 V scan in the potential range of -0.2~0.3 V.

**Figure S18.** XRD patterns of B-Sn-A after 3-h test in KHCO_3_, NaHCO_3_ and K_2_SO_4_.


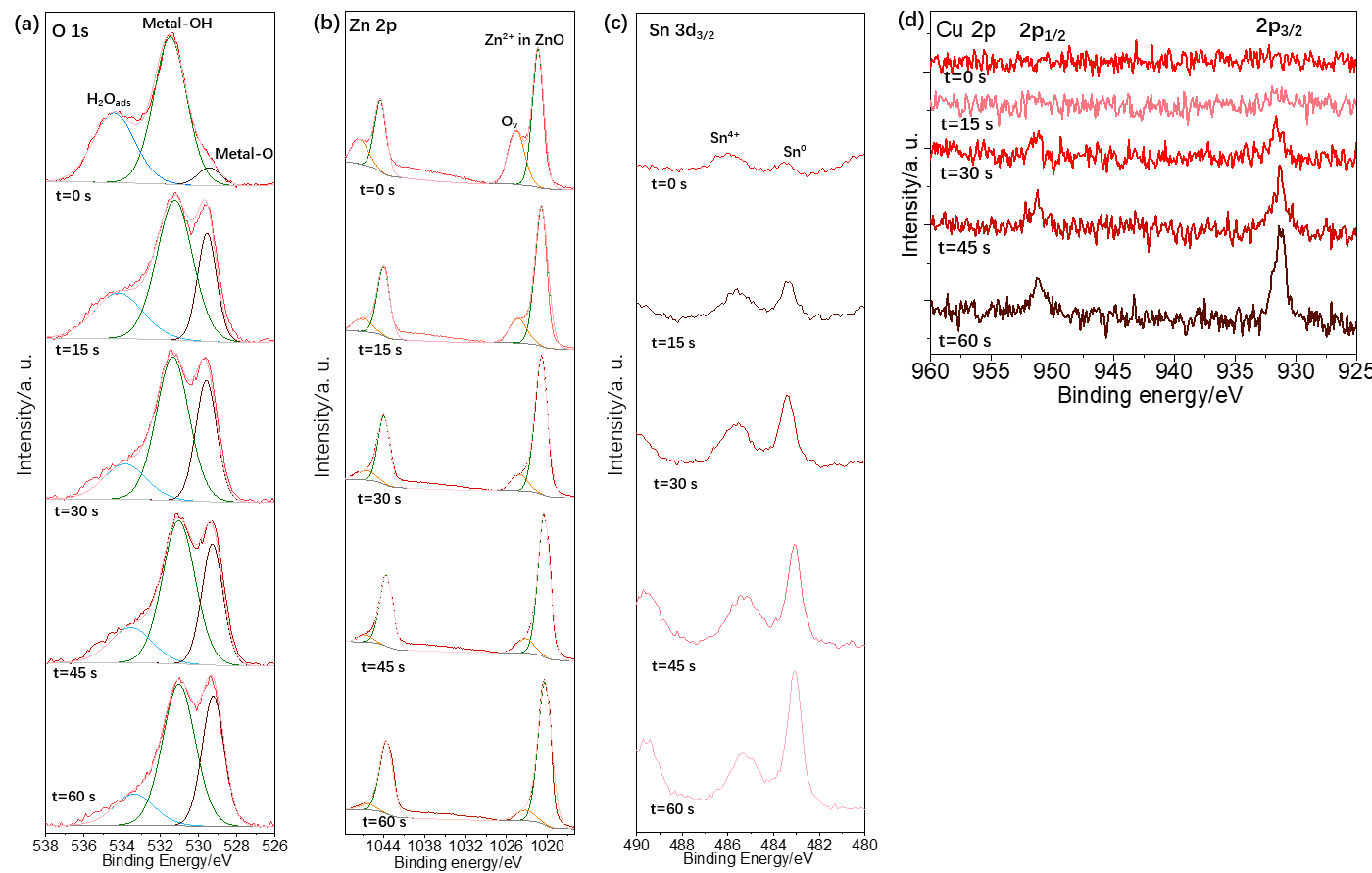


**Figure S19.** XPS spectra of B-Sn-A after 3-h test in KHCO_3_ by Ar^+^ sputtering: (a) O 1s, (b) Zn 2p, (c) Sn 3d_5/2_ and (d) Cu 2p.

The XPS O 1s spectra of B-Sn-A after 3-h test in KHCO_3_ show the metal-O and metal-OH signal, suggesting the metal oxide exist (Figure S19a).^[1, 6]^ The XPS Zn 2p spectra show the Zn^2+^ of ZnO and Zn^2+^ in vicinity of O vacancies (O_v_) (Zn-O_v_) exist.^[6]^ Interestingly, the intensity of Zn-O_v_ signal reduce with the sputtering time increasing, suggesting the O_v_ mainly exists on the surface (Figures 3c & S19b).^[7, 8]^ The XPS Sn 3d_5/2_ spectra show the existences of Sn^4+^ and Sn^0^, and the content of Sn^0^ increase with the depth increasing (Figures 3b & S19c).^[9-11]^ The Cu 2p peak intensities distribution suggest that the content of Cu decrease from bulk to surface (Figure S19d).


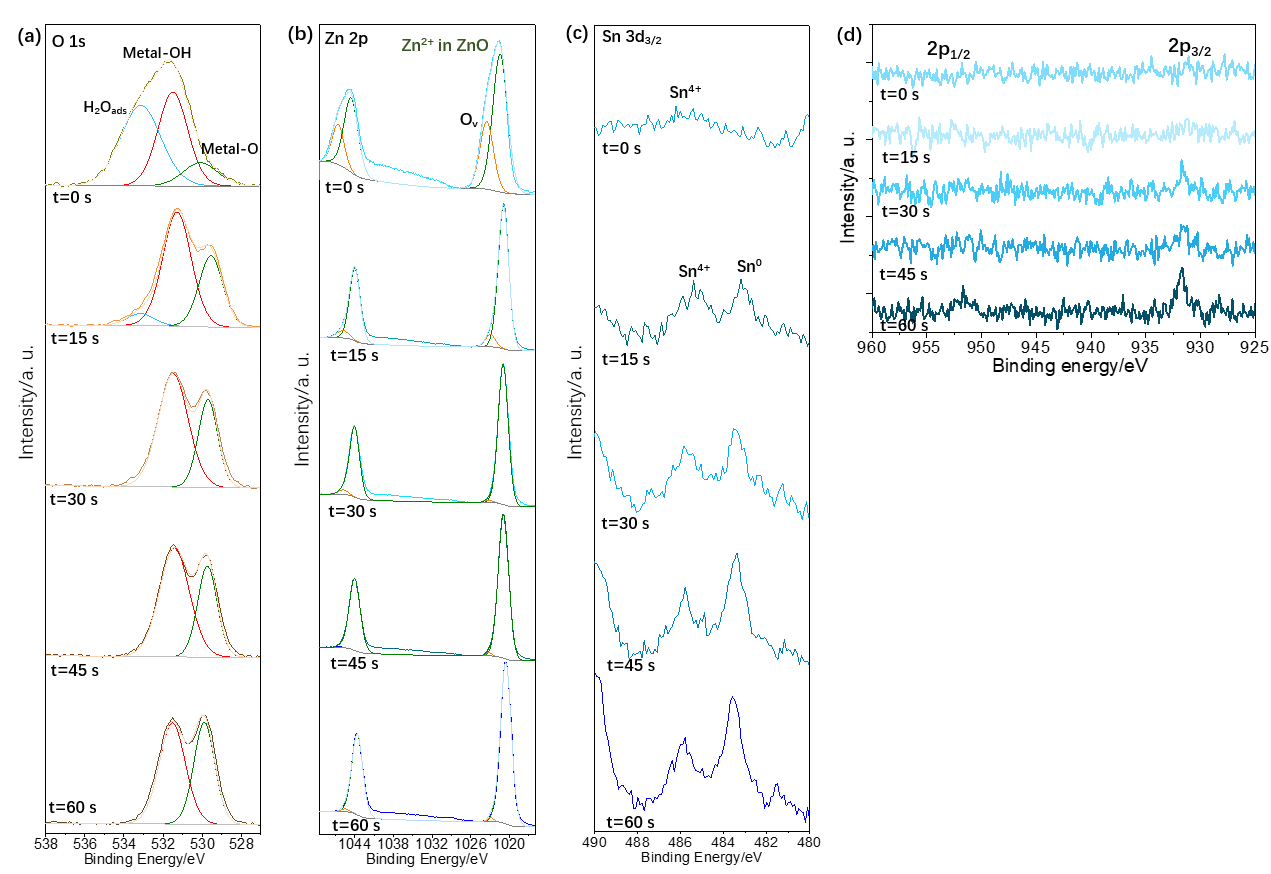


**Figure S20.** XPS spectra of B-Sn-A after 3-h test in NaHCO_3_ by Ar^+^ sputtering: (a) O 1s; (b) Sn 3d_5/2_; (c) Zn 2p; (d) Cu 2p.

The XPS O 1s spectra of B-Sn-A after 3-h test in NaHCO_3_ exhibit metal-O and metal-OH peaks, suggesting the existence of metal oxide (Figures S2a).^[1, 6]^ The XPS Zn 2p spectra show the existence of Zn^2+^ of ZnO and the Zn-O_v_, while the Zn-O_v_ mainly locates on the surface (Figures 3c & S20b).^[8]^ Meanwhile, XPS Sn 3d_3/2_ spectra suggest the existence of Sn^4+^ and Sn^0^,^[9-11]^ and the Sn mainly locates in the bulk and seldomly on the surface (Figures 3b & S20c). Moreover, the Cu 2p peak intensity decrease with the sputtering time decreasing, suggesting the density of Cu decrease from bulk to surface (Figure S20d).


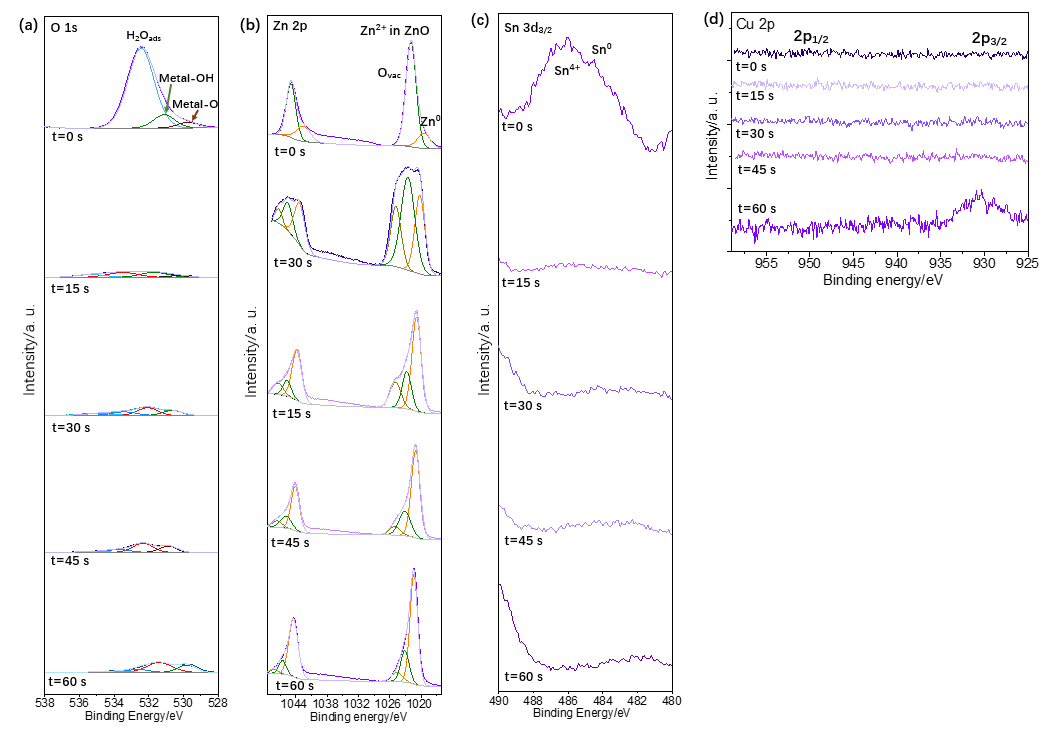


**Figure S21.** XPS spectra of B-Sn-A after 3-h test in K_2_SO_4_ by Ar^+^ sputtering: (a) O 1s, (b) Sn 3d_5/2_, (c) Zn 2p and (d) Cu 2p.

The XPS O 1s spectra of B-Sn-A after the 3-h test in K_2_SO_4_ exhibit metal-O and metal-OH peaks, indicating metal oxides (Figure S21a).^[1, 6]^ The peaks of Zn^2+^ in ZnO and Zn^0^ are observable. The ZnO signals reduce with the sputtering time increasing, suggesting ZnO locates on surface mainly (Figures 3c & S21b).^[4, 12]^ Interestingly, the Zn-O_v_ signal is negligible on the surface, but appears at 15 s then decrease from 15 to 60 s, demonstrating that the oxygen vacancies accumulate at sub-surface at a depth of ~7.5 nm (Figures 3c & S21b).^[8]^ The XPS Sn 3d_3/2_ spectra show that the Sn^4+^ and Sn^0^ signals are at the surface dominantly (Figure S21c).^[9, 10]^ The XPS Cu 2p spectra show that Cu is absent from the surface (Figures S21d).


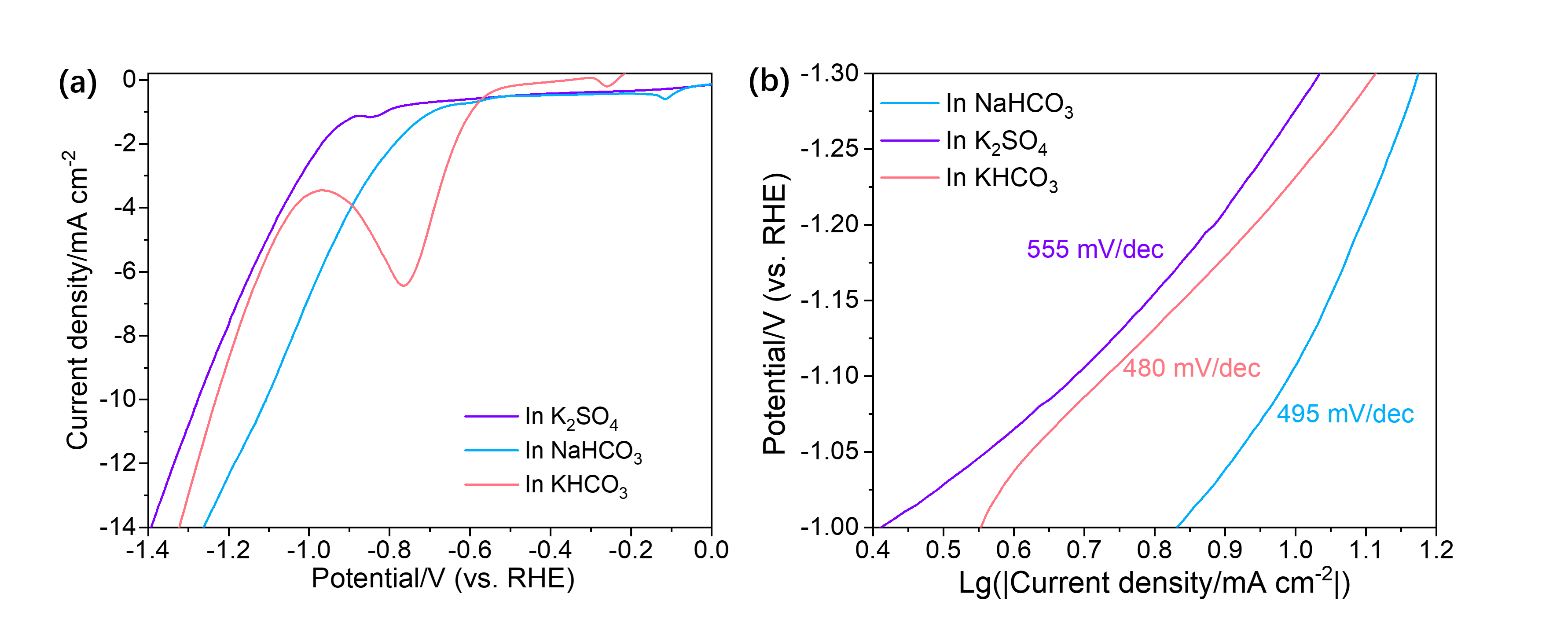


**Figure S22.** (a) LSV curves and (b) Tafel plots of B-Sn-A in KHCO_3_, NaHCO_3_ and K_2_SO_4_ without CO_2_.


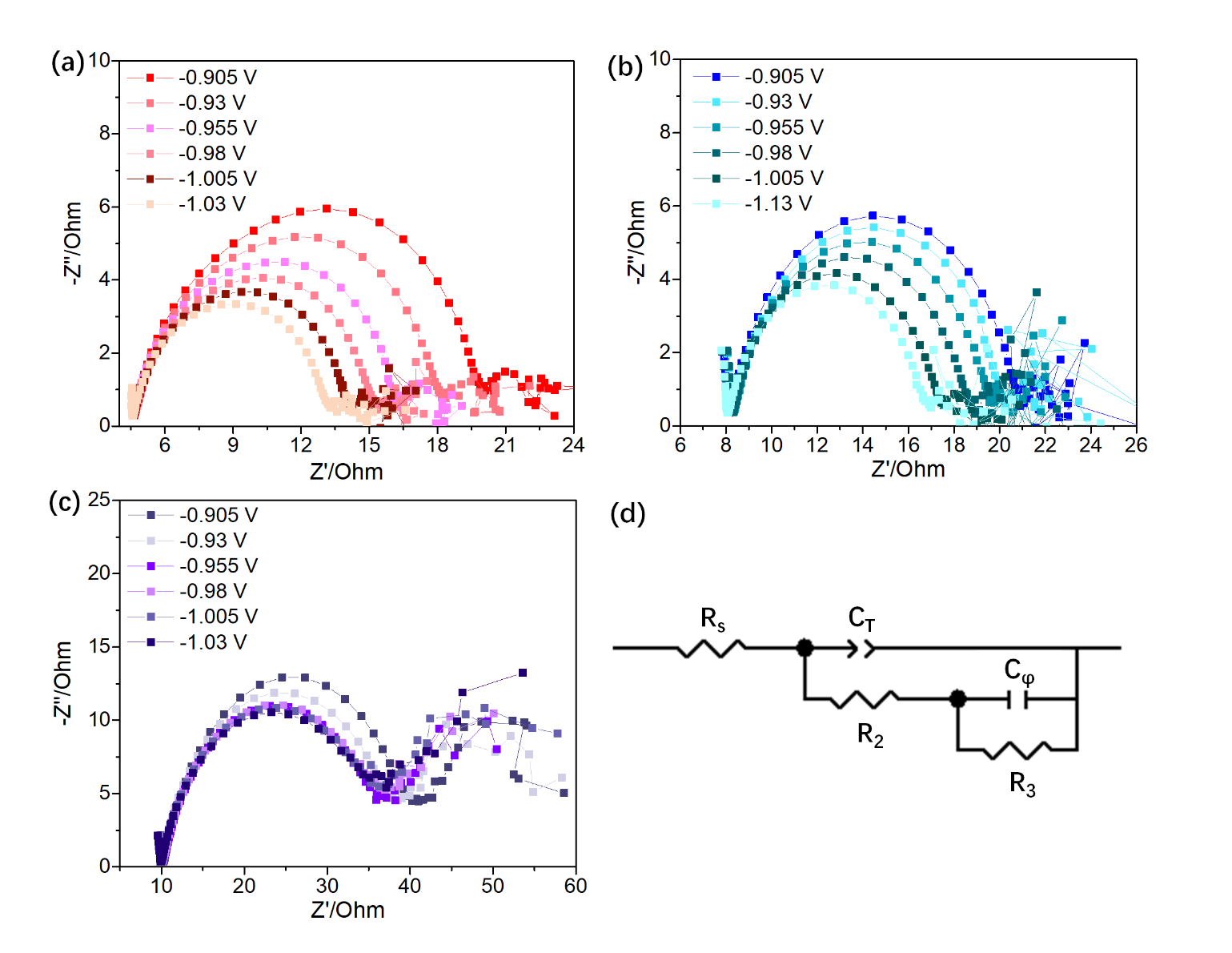


**Figure S23.** Nyquist plots of B-Sn-A after the 3-h test in: (a) 0.5 M KHCO_3_, (b) 0.5 M NaHCO_3_ and (c) 0.25 M K_2_SO_4_. (d) Electric circuit used to obtain the parameters.


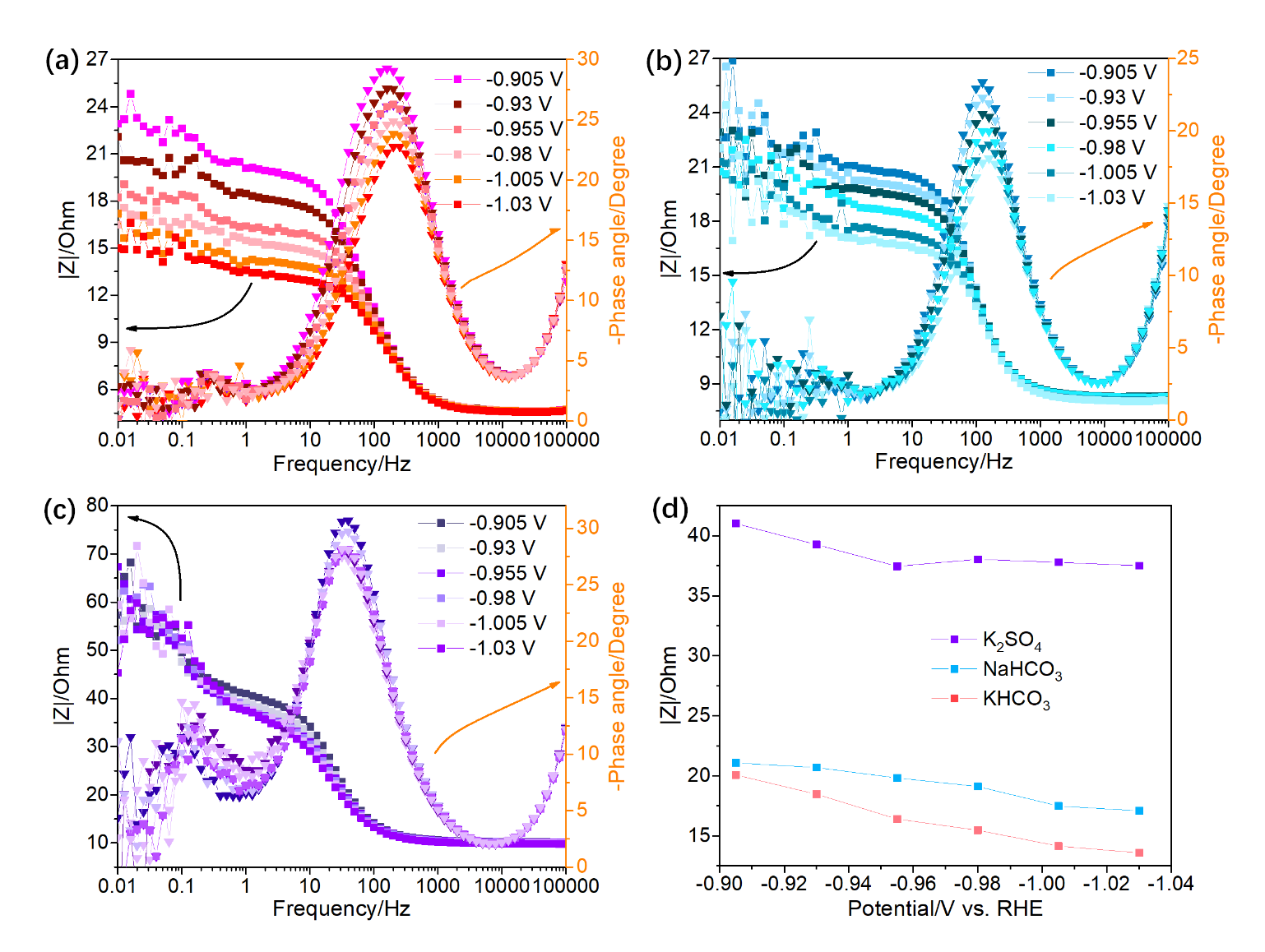


**Figure S24.** Bode plots of B-Sn-A after the 3-h test: (a) In 0.5 M KHCO_3_, (b) in 0.5 M NaHCO_3_, (c) in 0.25 M K_2_SO_4_, and (d) plots of |Z|-potential.

**Table S2.** The fitted parameters of the EIS data for B-Sn-A after the 3-h test in 0.5 M KHCO_3_, 0.5 M NaHCO_3_ and 0.25 M K_2_SO_4_.

| **Electrolyte** | **Potential/V vs. RHE** | **R_s_/Ω** | **C_T_/(F S^n^)^-1^** | **C_p_** | **R_2_/Ω** | **R_3_/Ω** | **C_φ_/F** |
| --- | --- | --- | --- | --- | --- | --- | --- |
| KHCO_3_ | -0.905 | 4.545 | 0.0005069 | 0.8146 | 15.81 | 2.513 | 0.3470 |
|  | -0.93 | 4.532 | 0.0005099 | 0.8203 | 11.92 | 2.068 | 0.3107 |
|  | -0.955 | 4.598 | 0.0005245 | 0.8182 | 10.83 | 1.914 | 0.3396 |
|  | -0.98 | 4.598 | 0.0005245 | 0.8182 | 10.83 | 1.382 | 0.3989 |
|  | -1.005 | 4.562 | 0.0004808 | 0.8327 | 9.671 | 2.32 | 0.3732 |
|  | -1.03 | 4.528 | 0.005224 | 0.8255 | 8.843 | 1.332 | 0.3792 |
| NaHCO_3_ | -0.905 | 8.176 | 0.000256 | 0.9240 | 12.71 | 1.899 | 0.2612 |
|  | -0.93 | 8.317 | 0.000265 | 0.9212 | 11.95 | 1.92 | 0.2713 |
|  | -0.955 | 8.329 | 0.000281 | 0.9130 | 11.31 | 1.951 | 0.3089 |
|  | -0.98 | 8.295 | 0.0002893 | 0.9112 | 10.42 | 1.545 | 0.1939 |
|  | -1.005 | 8.097 | 0.0003037 | 0.9074 | 9.41 | 2.317 | 0.2451 |
|  | -1.03 | 8.073 | 0.0003069 | 0.9076 | 8.75 | 1.276 | 0.2238 |
| K_2_SO_4_ | -0.905 | 10.28 | 0.0005541 | 0.8629 | 31.43 | 18.27 | 0.0997 |
|  | -0.93 | 10 | 0.0005897 | 0.858 | 29.27 | 11.07 | 0.066 |
|  | -0.955 | 10.1 | 0.0007207 | 0.8385 | 27.93 | 11.13 | 0.0479 |
|  | -0.98 | 10.11 | 0.0007130 | 0.8407 | 27.86 | 11.03 | 0.0473 |
|  | -1.005 | 9.972 | 0.0008028 | 0.8285 | 28.15 | 15.95 | 0.0512 |
|  | -1.03 | 9.878 | 0.0008866 | 0.8167 | 27.89 | 13.1 | 0.0475 |

**References**

[1] H. L. Pan, B. Yao, M. Ding, R. Deng, T. Yang, Y. R. Sui, T. T. Zhao, L. L. Gao, *J. Non-Cryst. Solids*, **2010**, *356*, 906-910.

[2] J.-H. Hong, Y.-F. Wang, G. He, J.-X. Wang, *J. Non-Cryst. Solids*, **2010**, *356*, 2778-2780.

[3] J. Winiarski, W. Tylus, K. Winiarska, I. Szczygieł, B. Szczygieł, *J. Spectrosc.*, **2018**, *2018*, 1-14.

[4] H. S. Jeon, J. Timoshenko, F. Scholten, I. Sinev, A. Herzog, F. T. Haase, B. Roldan Cuenya, *J. Am. Chem. Soc.*, **2019**, *141*, 19879-19887.

[5] T. Rakshit, I. Manna, S. K. Ray, *J. Appl. Phys.*, **2015**, *117*, 025704.

[6] P. T. T. Hoai, N. T. M. Huong, P. T. Huong, N. M. Viet, *Catalysts*, **2022**, *12*, 1194.

[7] A. T. T. Pham, D. V. Hoang, T. H. Nguyen, O. K. Truong Le, D. P. Wong, J.-L. Kuo, K.-H. Chen, T. B. Phan, V. C. Tran, *J. Alloys Compd.*, **2021**, *860,* 158518.

[8] G. Deroubaix, P. Marcus, *Surf. Interf. Anal.*, **2004**, *18*, 39-46.

[9] Y. Chen, M. W. Kanan, *J. Am. Chem. Soc.*, **2012**, *134*, 1986-1989.

[10] C. Yin, J. Hu, M. Cheng, T. Wei, Q. Liu, W. Li, Y. Ling, B. Liu, *Mater. Lett.*, **2022**, *314,* 131853.

[11] Y. Zhang, Z. Li, H. Jang, X. Wei, Q. Qin, X. Liu, *Inorg. Chem. Front.*, **2023**, *10*, 1818-1825.

[12] S. Tamiyakul, T. Sooknoi, L. L. Lobban, S. Jongpatiwut, *Appl. Catal. A*, **2016**, *525*, 190-196.
